# Technical efficiency of ghanaian health facilities before and during the COVID-19 pandemic

**DOI:** 10.1186/s12962-024-00575-8

**Published:** 2024-09-15

**Authors:** Gordon Abekah-Nkrumah, Charles Gyamfi Ofori, Maxwell Antwi, Alex Yao Attachey, Tobias F. Rinke de Wit, Wendy Janssens, James Duah, Charlotte Dieteren, Gifty Sunkwa-Mills

**Affiliations:** 1 Department of Health Services Management, University of Ghana Business School, Legon, P. O. Box 78, Accra, Ghana; 2 Department of Finance, University of Ghana Business School, Legon, P. O. Box 78, Accra, Ghana; 3 PharmAccess Foundation Ghana, East Cantonment, P.O. Box CT 10245, Accra, Ghana; 4 Amsterdam Institute for Global Health and Development (AIGHD), Vrije Universiteit Amsterdam, Paasheuvelweg 25, 1105 BP Amsterdam, The Netherlands; 5 Christian Health Association of Ghana, Labone, P. O. Box AN 7316, Accra, Ghana; 6 PharmAccess Foundation Amsterdam, Paasheuvelweg 25, 1105 BP Amsterdam, The Netherlands

## Abstract

**Purpose:**

Understanding the technical efficiency of health facilities is essential for an optimal allocation of scarce resources to primary health sectors. The COVID-19 pandemic may have further undermined levels of efficiency in low-resource settings. This study takes advantage of 2019 and 2020 data on characteristics of health facilities, health services inputs and output to examine the levels and changes in efficiency of Ghanaian health facilities. The current study by using a panel dataset contributes to existing evidence, which is mostly based on pre-COVID-19 and single-period data.

**Methods:**

The analysis is based on a panel dataset including 151 Ghanaian health facilities. Data Envelopement Analysis (DEA) technique was used to estimate the level and changes in efficiency of health facilities across two years..

**Results:**

The results show a net increase of 26% in inputs, influenced mostly by increases in temporary non-clinical staff (131%) and attrition of temporary clinical staff and permanent non-clinical staff, 40% and 54% respectively. There was also a net reduction in output of 34%, driven by a reduction in in-patient days (37%), immunization (11%), outpatients visits and laboratory test of 9%. Nowithstanding the COVID-19 pandemic, the results indicate that 59 (39%) of sampled health facilities in 2020 were efficient, compared to 48 (32%) in 2019. The results also indicate that smaller-sized health facilities were less likely to be efficient compared to relatively bigger health facilities.

**Conclusion:**

Based on the findings, it will be essential to examine factors that accounted for efficiency improvements in some health facilities, to enable health facilities lagging behind to learn from those on the efficiency frontier. In addition, the findings emphasise the need for CHAG to work with health facility managers to optimise inputs allocation through a redistribution of staff. Most importantly, the findings are suggestive of the resilience of CHAG health facilities in responding to a health shock such as the COVID-19 pandemic.

## Introduction

Globally, efficiency in health systems is a priority for decision-makers given that healthcare resources are scarce [1]. This is particularly important in regions such as Africa where resource scarcity in the health sector is acute. As such, Africa accounts for only 11% of the world’s population but carries 24% of the global burden of disease, and spends only 6.1% of its Gross Domestic Product (GDP) on health compared to 15% in developed countries [2–4]. Such resource scarcity in the midst of rising out-of-pocket expenditure, and the need to achieve financial protection and by extention Universal Coverage (UHC), reiterate the call for the health sector in Africa to be more efficient [5, 6]. Still, inefficient utilisation of healthcare resources persist in many African countries, with the average inefficiency rate estimated to be about 23% [7]. The inefficiency occurs as a result of misuse of health inputs in ways that do not maximise the level of health outputs produced (i.e. technical efficiency), or sub-optimal allocation of resources toward producing health outputs that are not priorities for society (allocative efficiency) [8].

However, given that health facilities consume the highest proportion of total health expenditure in many African countries (estimated to be from 45 to 81% of government health expenditure) [5, 9], they have inevitably become candidates to consider when it comes to efforts to understand and improve the levels of efficiency in the health sector. It has been suggested that efficiency in health systems, especially at the health facility level in Lower Middle Income Countries (LMICs), will not only help to ensure that limited available resources are used optimally, but can also result in savings that can be invested in the health system for improved population health [9, 10].

The existing literature abounds in peer-reviewed journal publications (40 papers identified) that have examined technical efficiency of health facilities in Africa [5]. Whiles existing studies used health facilities across different ownership types (public, mission, quasi public/ government and private) and hierarchy (health centres, hospitals etc.), they were mostly limited to using single-year data. In the specific case of Ghana, existing studies [11–15] are based on single-year data, making it difficult to examine trends and therefore changes in efficiency across years. Additionally, the only existing technical efficiency study in Ghana that includes mission health facilities is based on data collected in 2005 [12] and therefore can hardly be relied upon for policy decisions. The current study takes advantage of the Med4All baseline study, which collected data on inputs and output of Christian Health Association of Ghana (CHAG) health facilities for 2019 and 2020 to examine the level and changes in technical efficiency of CHAG health facilities for the two periods for which data was collected. Beside data availability, examining technical efficiency of CHAG health facilities is crucial given that it is the second largest provider of healthcare services in Ghana beside the Ghana Health Service, contributing an average of 30% of total OPD care from 2016 to 2021 [16]. CHAG facilities also serve majority of Ghana’s rural population. Most importantly, the unique nature of the Med4All data (data collected before and during the COVID-19 pandemic) makes it possible to examine whether CHAG health facilities were resilient enough to withstand shocks emanating from the COVID-19 pandemic. Specifically the paper examines:

1. Level and determinants of technical efficiency among CHAG health facilities
2. Changes in technical efficiency of CHAG health facilities between 2019 and 2020.

The current study is essential in terms of added value in two respects. First, the findings affords us the opportunity to know the level of improvements achieved by CHAG health facilities in technical efficiency compared to when they were last studied using 2005 data [12]. Secondly, a comparison of efficiency scores for 2019 and 2020 makes it possible not only to identify changes that occurred between the two periods, but more importantly, whether the shock arising from the COVID-19 pandemic adversely affected the levels of efficiency of CHAG health facilities or not.

## Methods

The study used health service input and output data from the Med4All baseline study, details of this project are described elsewhere [17]. The Med4All study, which has completed baseline data collection and awaiting endline data collection in 2024 seeks to examine the impact of a health facility-based medicines supply chain innovation (Med4All system) on the efficiency of participating health facilities. The baseline data was collected from 151 CHAG health facilities out of a universe of 316 yet to use the Med4All system. The sample of 151 health facilities was arrived at first, by calculating a sample size deemed to be large enough to isolate the impact of the implementation of the Med4All system on efficiency of participating health facilities. This was done via power calculations that used a design effect of 7% based on the existing literature [18–20] and standard deviation of 23.3% as per Jehu-Appiah etal. [12] for mission hospitals in Ghana, an alpha value of 0.05 and default power of 0.8. The power calculations resulted in a an optimal sample size of 119 health facilities to be used for the Med4All study. However, to take care of sample attrition, the sample size of 119 was increased to 151.

The Med4All baseline data was collected using a digitized data collection instrument via the KoboCollect software. For each sampled health facility, data supervisors worked with enumerators recruited from health facilities to collect and enter data on variables of interest (in the case of the current paper: service input and output and characteristics of health facilities) for the years 2019 and 2020 into a data repository. Data on each health facilitiy entered into the data repository was later sent back to senior officials of respective health facilities for validation. Additionally, data collected on each health facility was validated with a version of the same data submitted by each health facility as part of routine report to CHAG. For the purpose of the current study, specific variables used are captured in Tables 1 and 2 below. Note that there was no attrition of health facilities between 2019 and 2020.

**Table 1:** Inputs and outputs used for the study

Inputs	Outputs
Total number of temporary clinical staff	Total outpatient visits
Total number of permanent clinical staff	Total inpatient days
Total number of temporary non-clinical staff	Total number of ANC visits
Total number of permanent non-clinical staff	Total number of deliveries
Total number of beds	Total number of children immunized
Total expenditure	Total number of laboratory tests

**Table 2:** Characteristics of health facilities; *Source:* Auhtors’ calculation based on field data

Item	Number of facilities	Percentage
*Type of Facility*		
Primary Care Hospitals	69	45.7%
Secondary Care Hospitals	6	4%
Other Lower-Level Facilities	76	50.3%
*Gender of CEO*		
Male	111	73.5%
Female	38	25.2%
Non response	2	1.3%
*Educational level of CEO*		
Secondary	2	1.3%
Diploma	13	8.6%
Bachelor	76	50.3%
Masters	56	37.1%
Doctorate	2	1.3%
Non response	2	1.3%
*Average age of facility*		
2019 avg. age	29.2 years	
2020 avg. age	30.2 years	
*Average age of CEO*		
2019 average age	42.97 years	
2020 average age	43.97 years	

### Analysis

A two-stage process was used for the analysis. In the first stage, Data Envelopement Analysis (DEA) technique was used to seperately calculate efficiency scores for the 151 health facilities as well as changes in the scores over the 2 years. The DEA technique was also used to compute output improvements needed for inefficient health facilities to become efficient using the 2020 data. The methods are further explained in the paragraphs below.

### Determination of inputs and outputs

The determination of inputs and outputs for a group of decision making units (DMUs) is an important aspect of the DEA technique. In the health sector, the main output for healthcare facilities is improved health status [11]. However, proxies are used in measuring the output of improved healthcare since it is difficult to explicitly measure this variable [11, 12, 21]. In a typical health facility, input categories will include human resources (clinical and non clinical staff), equipments (Number of beds, spending on relevant assets), medicines and supplies (spending on medicines and consumerbles etc.) and Land (floor space). Output however includes categories such as preventive and promotive services (immunisation, antenatal care and health outreach), curative care (outpatients care, inpatients care, deliveries, surgeries etc.) and anxcillary services (laboratory test, imaging etc.). In line with these proxies and availability of data in the Med4All dataset, we selected human resources (number of clinical and non-clinical staff) and capital (number of beds, general expenditure etc.). Information on floor space was however not available. In the case of input, we selected preventive and promotive services (immunisation, antenatal visits), curative services (outpatients, inpatients visits and number of deliveries) and axcillary services ( number of laboratory test) as per Table 1.

### Efficiency and the DEA model

Efficiency which measures the relative performance of DMUs to the best performing DMUs on the production possibility frontier can either be technical or allocative [22]. Technical efficiency (focus of this study) is the capacity of a DMU to use the minimum set of inputs to achieve a certain level of output (input-oriented) or produce the maximum set of outputs with a given level of inputs (output-oriented).

Unlike parametric approaches that relies on economic theory, DEA which was first proposed by Farrell [22], measures relative efficiency through a linear programming technique and is guided by data to determine the location and shape of the efficiency frontier. The original model was further developed (i.e. CCR model) to assume constant returns to scale (CRS) and therefore capture sensitivity in measuring technical efficiency [23]. Intuitively, CRS means that a percentage change in inputs results in the same percentage change in outputs. However, this is implausible in real market situations due to imperfect market conditions and regulatory changes. Thus, the DEA model was further developed (i.e. the BCC model) to assume variable returns to scale (VRS).

The CCR model determines the gross efficiency of the DMU by measuring the ability of the DMU to convert its inputs into outputs. On the other hand, the BCC model decomposes technical efficiency into Pure Technical Efficiency (PTE) and Scale Efficiency (SE) [24], which is related to the size of the DMU. There are two types of SEs in an inefficiency situation. These are decreasing returns to scale (DRS), which reflect a situation where the size of a DMU may be too large for its activities and therefore results in diseconomies of scale. Increasing returns to scale (IRS) on the contrary reflect a situation where the DMU is too small for the size of its operations and therefore leads to economies of scale. Under the VRS or CRS approach, relative efficiency can be measured using the input-oriented or output-oriented approach. In DEA, each DMU can choose any combination of inputs and outputs to maximise efficiency, which technically is the ratio of total weighted output to total weighted input. Thus, DMUs that employ less input to produce more outputs are deemed to be on the efficiency frontier’s edge (i.e. efficient) and therefore have an efficiency score of 1 or 100%. On the other hand, DMUs within the production possibility set are regarded as inefficient with a score of less than 1 or 100%. DEA's main advantage is its ability to utilise multiple inputs and outputs and its simplicity in interpretation. For a detailed discussion of the advantages and limitation of the use of DEA refer to Jehu-Appiah et al. [12].

For this study, we consider N health facilities (or DMUs) where each health facility utilises $x$ inputs and $y$ outputs, where ${x}_{i}$ represent the $i\text{th}$ input and ${y}_{j}$ represent the *j*th output. The output oriented CCR model is described by the following linear programming model:

1a $$Max\, {E}_{m}=\sum_{j=1}^{J}{a}_{jm}{y}_{jm}$$

Subject to

1b $$\begin{aligned}& \sum_{j=1}^{J}{b}_{jm}{x}_{jm}=1\\ &\sum_{j=1}^{J}{a}_{jm}{y}_{jn}-\sum_{j=1}^{J}{b}_{jm}{x}_{jn}\le 0;\forall n\\ & {a}_{jm},{b}_{jm}\ge 0; \forall i,\forall j\end{aligned}$$

This study uses the BCC output-oriented approach, because inputs are limited and health facility managers are not able to change that in the short-run in lower-middle income countries like Ghana. Thus, it is more reasonable for health facility managers to focus on maximising output given the set of existing inputs. Additionally the BCC model makes it possible to decompose computed efficiency scores into pure technical efficiency and scale efficiency. The variable ${E}_{m}$ represents the virtual output of the health facility $m,$ which is a linear combination of all the outputs of the health facility, where each output is given the weight ${a}_{j}$. The objective function seeks to choose weights ${a}_{j}$ for health facility $m$ that maximises its outputs. The output-oriented model seeks to maximise the output given the set of input levels. Thus, the linear programming model constraints the linear combination of inputs for health facility $m$ to be equal to 1, as shown in the first part of Eq. 1b. The quantity ${b}_{jm}$ represents the weight for input $j$ for health facility $m.$ The second part of the constraint indicate that for each DMU, the difference between the weighted sum of all outputs and the weighted sum of inputs of all the DMUs is less than or equal to zero. This constraint allows the respective input and output weights ${a}_{jm}$ and ${b}_{jm}$ to be selected such that the efficiencies of other DMUs are restricted to lie within the closed interval of 0 and 1.

Under the VRS approach, the output oriented CCR multiplier model is modified by introducing the quantity ${z}_{jm}$ to the objective function and constraints. The health facility or DMU exhibits increasing returns to scale if ${z}_{jm}>0$ and decreasing returns to scale if ${z}_{jm}<0$. The health facility experiences a constant returns to scale if ${z}_{jm}=0$. The BCC model is provided by the following equations.

2a $$Max\, {E}_{m}=\sum_{j=1}^{J}{a}_{jm}{y}_{jm}+{z}_{jm}$$

Subject to

2b $$\begin{aligned}&\sum_{j=1}^{J}{b}_{jm}{x}_{jm}+{z}_{jm}=1\\ & \sum_{j=1}^{J}{a}_{jm}{y}_{jn}-\sum_{j=1}^{J}{b}_{jm}{x}_{jn}+{z}_{jm}\le 0;\forall n\\ & {a}_{jm},{b}_{jm}\ge 0; \forall i,\forall j\end{aligned}$$

It is important to emphasise that traditional DEA approach can be susceptible to potential bias in the estimation of efficiency scores due to outliers and statistical noise. To address this, the study employed the Simar and Wilson bootstrapping method. This approach provides bias corrected scores with an accompanying 95% confidence interval. However, our assessment show that the distribution of the traditional efficiency scores mimics that of the computed bias corrected scores. This is also shown in the Kendall’s rank correlation coefficient which shows a substantial level of association between the ranks for the traditional efficiency scores and the bias corrected scores (See Table AP-2.1 of Appendix 2). Turkson and colleagues [25] explain that a consistent mirroring of traditional and bias corrected efficiency scores provide additional justification that the traditional efficiency scores are not subject to the risk of potential bias and outliers. Secondly, unlike the traditional efficiency scores, it is difficult to decompose the bias corrected scores into respective scale efficiencies and also assess returns to scale under scale efficiency. Thus, given the added value to the study of scale efficiency and returns to scale, the traditional efficiency scores were used. Notwithstanding, the bias corrected scores and respective confidence intervals are provided in Tables AP-2.2 to AP-2.4 in Appendix 2.

In addition to the efficiency scores, The DEA model creates an efficiency frontier where efficient DMUs who achieve the largest possible outputs for their inputs lie on the frontier (under the output-oriented model). Conversely, DMUs, (i.e. health facilities), that are inefficient do not lie on the frontier. Efficient DMUs that share similar input–output characteristic with their inefficient DMUs can act as peers. Thus, certain efficient DMUs could be used as benchmarks for inefficient DMUs. Mathematically, the DEA model determines the peers for each inefficient DMU by solving the dual of the primal envelopment model (See Ramanathan [26] for more information on the mathematical solution of the primal and dual problems). Following from the model properties, this study proceeded to identify the peers for each inefficient DMU in order to estimate the potential improvement in output required for inefficient DMUs to become efficient.

## Results

### Health facility characteristics

Data was collected among 151 CHAG health facilities, of which half of the sample (50.3%) constituted of lower-level health facilities, slightly less than half (45.7%) were primary care hospitals and the rest (4%) were secondary care hospitals. Sampled health facilities have been in existence for a minimum of 1 year and a maximum of 71 years, with an average of 29 years in 2019 and 30 years as of 2020. In addition, majority of health facility CEOs were males (74%), with majority of them having either a bachelor’s degree (50.3%) or a masters degree or higher (39.7%). Table 2 provides summary characteristics of health facilities studied.

### Input and output characteristics

Health facilities surveyed had a minimum of 2 and a maximum of 357 beds, with an average of 56 beds for 2019 and 2020. Total expenditure including salaries increased from GHS 661 million in 2019 to about GHS 727 million in 2020, with the average increasing from GHS4.38 million to GHS4.81 million for the same period. However, average total expenditure exclusive of salaries for 2020 reduced by 9% from the 2019 figure of GHS 1.67 million. In 2019, 122 out of the 151 health facilities sampled had at least one temporary clinical staff, but reduced by 4% to 117 facilities in 2020. Temporary clinical staff reduced by 40% from 1679 in 2019 to 1015 in 2020. The number of permanent clinical staff also dropped by 2% from 13,890 in 2019 to 13,650 in 2020. On the contrary, the number of temporary nonclinical staff increased significantly between 2019 (855) and 2020 (1974). The total number of permanent non-clinical staff on the other hand reduced by 54% between 2019 and 2020.

For output, the average number of inpatient and outpatient visits reduced by 37% and 9% respectively between the 2 years. Average number of ANC visits increased by 2%, while the average number of deliveries per health facility increased by 8%. The average number of children immunized increased by 11%, whereas laboratory tests reduced by 9%. Table 3 presents summary statistics of inputs and output variables used.

**Table 3:** Input and output characteristics of DMUs; *Source:* Auhtors’ calculation based on field data

Item	2019 (n)	2020 (n)	% Change
*Inputs*			
Beds	56	56	0%
Average Expenditure (Incl salary in GHS Million)	4.38	4.81	9.8%
Average Expenditure Excl of salary (GHS Million)	1.67	1.51	− 9%
Total Temporary clinical staff	1679	1015	− 40%
Total Permanent clinical staff	13,890	13,650	− 2%
Total Temporary non-clinical staff	855	1974	131%
Total Permanent non-clinical staff	4178	1902	− 54%
*Outputs*			
In-patient days	13,980.11	8865.22	− 37%
outpatient visits	31,043.49	28,153.34	− 9%
ANC	3443.49	3502.53	2%
Children immunized	4451	4926	11%
Deliveries	657	707	8%
Lab tests	46,572	42,335	− 9%

### Analysis of efficiency scores

The technical efficiency and related decomposed efficiency scores in 2020; pure technical and scale efficiencies, were obtained from the inputs and outputs identified in the DEA model. The results of the DEA analysis (see Table 4) show an average Technical Efficiency (TE) score of 0.73 with a standard deviation of 0.28. Also, 59 health facilities, representing 39% of the sample, were technically efficient, thus having a TE score of 1.

**Table 4:** Efficiency scores and frequency in 2020 (N = 151) *Source*: Authors’ calculation based on field data

Efficiency type	Mean	SD	Min	Max	% of Sample
*Summary Efficiency Scores*					
Technical Efficiency	0.74	0.28	0.01	1	39%
Pure Technical Efficiency	0.79	0.27	0.03	1	49%
Scale Efficiency	0.92	0.11	0.41	1	39%

Efficiency by Health Facility Type					Health Fac. Of Frontier
*Primary Care Hospitals N = 69*					
Pure Technical Efficiency	0.81	0.23	0.34	1	31 (44.9%)
Scale Efficiency	0.88	0.12	0.61	1	22 (31.9%)
Overall Technical Efficiency	0.72	0.26	0.27	1	22 (31.9%)
*Secondary Care Hospitals N = 6*					
Pure Technical Efficiency	0.96	0.06	0.87	1	4 (66.7%)
Scale Efficiency	0.90	0.12	0.71	1	3 (50%)
Overall Technical Efficiency	0.87	0.09	0.71	1	2 (33.3%)
*Other Lower Level Facilities N = 76*					
Pure Technical Efficiency	0.77	0.26	0.03	1	39 (51.3%)
Scale Efficiency	0.95	0.12	0.41	1	42 (55.3%)
Overall Technical Efficiency	0.74	0.31	0.01	1	35 (46.1%)

Overall, Scale Efficiency (SE) and Pure Technical Efficiency (PTE) scores after decomposition were 0.92 and 0.79 with standard deviations of 0.11 and 0.27 respectively (see Table 4).

**Fig. 1:**
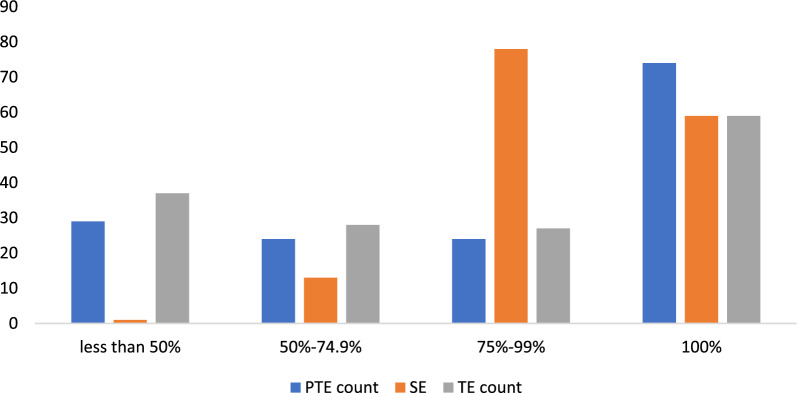
Distribution of Efficiency Scores in 2020. Source: Constructed by authors based on field data

The low dispersion in the distribution of individual SE scores contributed to its high average scores. The SE scores recorded the lowest standard deviation, which shows that most of the SE scores for the health facilities were not highly dispersed away from the mean. Again, Fig. 1 shows that a large proportion of health facilities sampled had higher SE scores, although these were not 1. For instance, 78 health facilities (representing 52%) had SE scores between 0.75 and 0.99, compared with 24 health facilities (representing 16%) and 27 health facilities (representing 18%) with respective PTE and TE scores ranging between 0.75 and 0.99.

Table 4 equally summarises efficiency scores based on the health facility type (Primary Care Hospitals—PCH, Secondary Care Hospitals—SCH, and Other Lower Level Health Facilities—OLHF—i.e. Clinics, CHPS Compounds and Health Centres). SCH recorded the highest average PTE score of 0.96, while PCH followed with an average PTE score of 0.81. OLHF recorded the least PTE score of 0.77, but the highest average SE score of 0.95, followed by SCH with an average SE score of 0.90 and PCH with the least SE score of 0.88. OLHF recorded the second-highest TE score of 0.74, driven mostly by a higher SE score. PCH however recorded the least TE score of 0.72.

Again, Table 4 shows that a substantial proportion of SCH were on the efficiency frontier (i.e. TE score of 1). Wheras 66.7% and 50% of SCH had a PTE and SE score of 1 respectively, only 2 (33.3%) SCH had a TE score of 1. For PCH’s, 31(44.9%) and 22 (31.9%) facilities respectively had PTE and SE scores of 1, with the number of PCH’s having a TE score of 1 being 22 (31.9%). For the OLHF sample, 51.3% (39) and 55.3% (42) had a PTE and SE score of 1 respectively, with 46.1% of the sample recording a TE of 1. This implies that PCHs had a lower proportion of their sample on the efficiency frontier.

#### Assessment of returns to scale

Table 5 presents the proportions of health facilities that exhibit constant, increasing and decreasing returns to scale according to the type of health facility. The results show that the OLHF had the highest proportion of health facilities exhibiting CRS (46%), followed by SCH (33%) and PCH (32%). Comparatively, PCH had the highest proportion of health facilities exhibiting DRS (65%), whereas OLHF had the highest proportion of health facilities experiencing IRS (36%).

**Table 5:** Returns to scale assessment for sampled health facilities in 2020; *Source*: Authors’ calculation based on field data

Facility type	CRS	DRS	IRS	N
Primary Care Hospital	22 (32%)	45 (65%)	2 (3%)	69
Secondary Care Hospital	2 (33%)	3 (50%)	1 (17%)	6
Other Lower Level Health Fac	35 (46%)	14 (18%)	27 (36%)	76
**Grand Total**	**59(39%)**	**62(41%)**	**30(19.9%)**	**151**

#### Potential output improvement by the type of health facility

The DEA model was used to compute general efficiency scores of health facilities. Subsequently, it was used to identify possible output improvements needed for inefficient health facilities to be efficient. This section outlines improvements in the various output variables used in computing the efficiency scores.

**Fig. 2:**
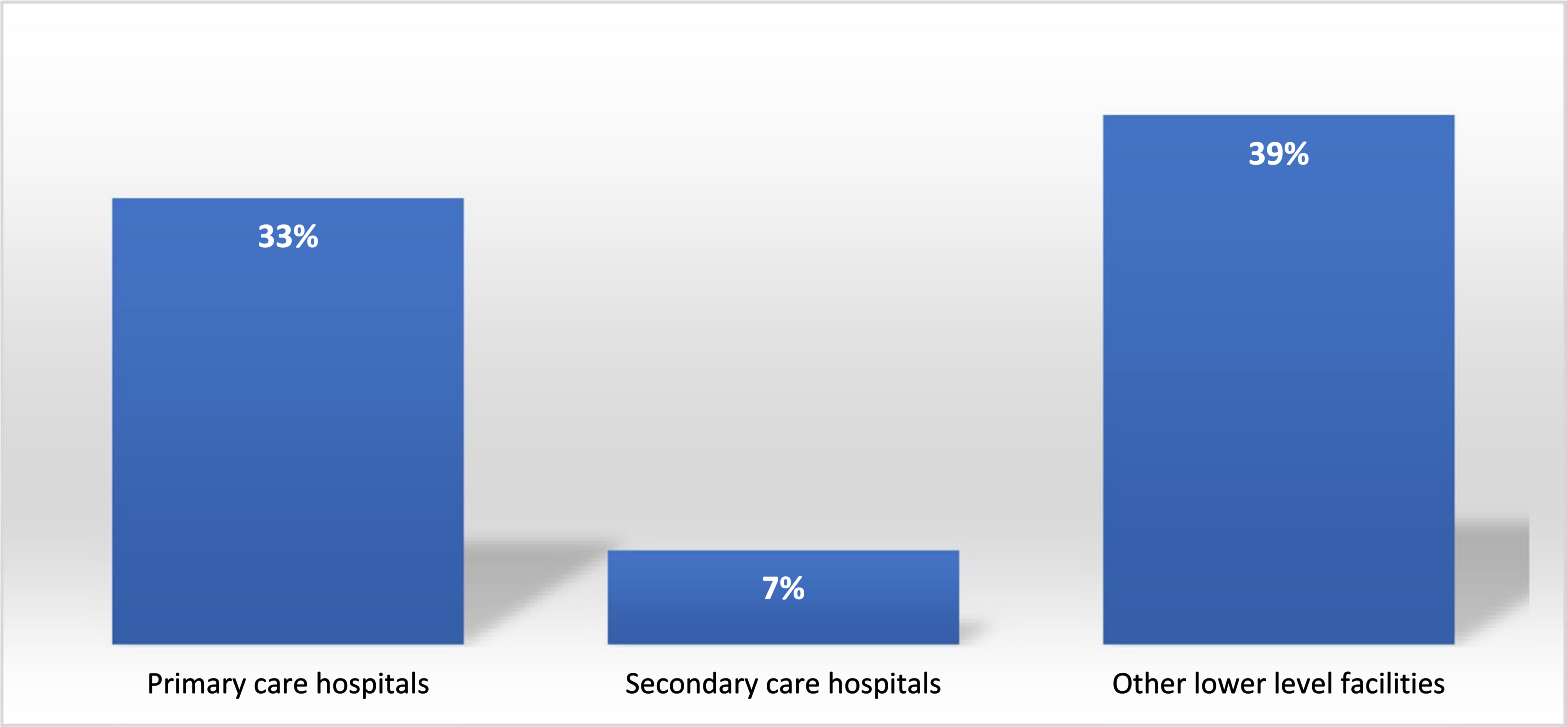
Average output improvement required to reach efficiency by health facility type. Source: Authors' calculation based on field data

**Fig. 3:**
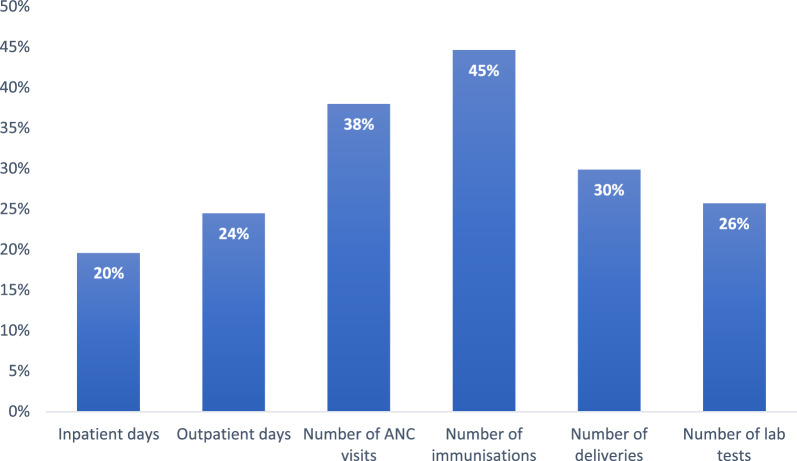
Average output improvement required to reach efficiency by output type. Source: Authors’ calculation based on field data

As per Fig. 2 the highest level of improvement required for efficiency emerged from OLHF, followed closely by PCH. The DEA model recommends that the sampled health facilities within OLHF need to improve the specified outputs by 39% to ensure 100% efficiency. On the other hand, PCH require an improvement of about 33%, while SCH require an improvement of about 7% in their specified outputs. Generally, the biggest improvement required for efficiency is related to the number of immunizations (see Fig. 3). The model required that immunizations must be improved by about 45%, followed by the number of ANC visits which required improvements of 38%. Outputs requiring the least level of improvement were inpatient days (20%), outpatient days (24%) and the number of laboratory tests (26%).

As per Table 6, further analysis was conducted to estimate expected improvement (i.e. by output and type of health facility) required for health facilities to operate at the frontier.

**Table 6:** Potential output improvement per type of health facility; Source: Authors’ calculation based on field data

	Actual	Projection	Difference	% change
*Primary Care Facilities*				
Inpatient days	15,257.51	18,428.93	3171.42	21%
Outpatient days	43,701.74	54,529.71	10,827.97	25%
Number of ANC visits	5135.50	7402.40	2266.90	44%
Number of immunizations	6665.31	10,011.79	3346.48	50%
Number of deliveries	1132.29	1515.98	383.69	34%
Number of lab tests	73,132.64	92,012.31	18,879.68	26%
*Secondary Care Facilities*				
Inpatient days	33,206.22	34,313.78	1107.56	3%
Outpatient days	85,504.89	90,913.07	5408.18	6%
Number of ANC visits	12,084.22	12,480.13	395.91	3%
Number of immunizations	13,620.44	14,862.04	1241.60	9%
Number of deliveries	2557.11	2688.92	131.81	5%
Number of lab tests	118,812.89	136,232.74	17,419.85	15%
*Other Lower Level*				
Inpatient days	1140.04	1621.72	481.68	42%
Outpatient days	9509.28	12,922.57	3413.29	36%
Number of ANC visits	1342.46	1893.89	551.43	41%
Number of immunizations	2661.23	3887.75	1226.52	46%
Number of deliveries	175.25	235.73	60.48	35%
Number of lab tests	8336.63	11,407.17	3070.55	37%

For PCH, immunization emerged as the output requiring the most improvement (50%), followed by deliveries and laboratory tests, requiring 34% and 26% improvement respectively, outpatient visits requiring 25% improvement and average inpatient days, requiring a 21% improvement to ensure that such facilities are placed on the efficiency frontier.

The results also suggest that SCH generally require lower levels of output improvement to be efficient. The number of laboratory tests emerged as the output needing the most improvement (15%). This was followed by the need for 9%, 6%, 5%, 3% and 3% improvements/increase in immunization, outpatient days, deliveries, ANC visits and inpatient days respectively.

The most significant improvement required for lower level health facilities came from the number of immunizations. The model require that lower level health facilities improve their number of immunizations by 46% from the current value of about 2600 to about 3800. The number of inpatient and outpatient days were also to be improved by 42% and 32%, respectively. Lower level health facilities were also required to improve ANC visits, deliveries and laboratory tests by 41%, 35% and 37% respectively. Analysis of output improvement for individual health facilities is provided in Table AP-1.3 in the Appendix.

### Changes in efficiency scores of health facilities

As per Table 7, Overall TE improved significantly by 11.1% from 2019 to 2020 $\left(t=4.14;p=0.00\right).$ The analysis shows that overall TE was generally impacted mainly by changes in SE. The average SE increased significantly by about 11.5% between 2019 and 2020 $(t=6.94;p=0.00)$. On contrary, there was no significant increase in PTE between 2019 and 2020 $(t=0.9,p=0.18)$. Table 8 further indicates an improvement in the number of efficient health facilities. For instance, purely technical efficient (i.e. PTE) and technically efficient (i.e. TE) health facilities increased by 15% and 11% respectively between 2019 and 2020. There was however no substantial change in the number of scale-efficient health facilities.

**Table 7:** Changes in efficiencies of health facilities; *Source:* Authors’ calculation based on field data

	2019	2020	%Change	p values*
*Panel A: Average Efficiency Scores*				
Technical efficiency	0.665	0.739	11.1%	0.00
Pure technical efficiency	0.788	0.791	0.4%	0.18
Scale efficiency	0.826	0.920	11.5%	0.00
*Panel B: Percentage of Sampled Health facilities Exhibiting Efficiency of 100%*				
Technical efficiency	48(31.8%)	59(39.15%)	11%	
Pure technical efficiency	52(34.4%)	67(44.4%)	15%	
Scale efficiency	74(49%)	74(49%)	0%	

* This results is from a paired sample t-test determining the significance of the difference in efficiency scores between 2019 and 2020. See Tables 11 and 12 for detailed scores for all health facilities in the Appendix

**Table 8:** Changes in technical and scale efficiency by facility type; *Source:* Authors’ calculation based on field data

Health facility type	Efficiency scores					
	TE		PTE		SE	
	2019	2020	2019	2020	2019	2020
*Efficiency scores*						
Primary Care Hospital	0.614	0.723	0.777	0.805	0.768	0.885
Secondary Care Hospital	0.641	0.866	0.939	0.964	0.683	0.901
Other Lower Level Fac	0.714	0.743	0.786	0.765	0.889	0.954
*Number and % of Efficient Health Facilities*						
Primary Care Hospital	(13)19%	(22)32%	(29)42%	(30)43%	(13)19%	(22)32%
Secondary Care Hospital	(1)17%	(2)33%	(3)50%	(4)67%	(1)16%	(2)33%
Other Lower Level Health Fac	(34)44%	(35)46%	(41)54%	(37)49%	(34)44%	(35)45%

Table 8 summarises efficiency scores based on health facility type. The average TE score for PCH improved by about 18%, whereas their PTE scores improved by about 4%. SE scores for PCH also improved by 15%, from 0.77 in 2019 to 0.88 in 2020. Secondary Care Hospitals, on the other hand, witnessed a substantial increase in overall TE scores by about 35. This increase was largely contributed by the significant increase in their SE scores (from 0.68 in 2019 to 0.90 in 2020, representing about 32% increase). OLHF improved their TE scores by about 4% but had a marginal reduction in PTE scores by about 3%. SE scores for lower level health facilities also improved by about 7%, from 0.89 in 2019 to 0.95 in 2020.

Table 9 provides changes in returns to scale between 2019 to 2020. The results show that in 2019 about 48 health facilities, representing approximately 32%, were scale efficient, exhibiting constant returns to scale. In 2020, the sample’s proportion of scale-efficient health facilities increased by 23% to 59 health facilities. Conversely, health facilities exhibiting decreasing returns to scale reduced from 80 to 62 in 2020. For these two years, health facilities experiencing DRS formed the majority. Health facilities exhibiting IRS increased by about 30%, from 23 health facilities in 2019 to 30 in 2020.

**Table 9:** Returns to scale assessments for sampled health facilities; *Source*: Authors’ calculation based on field data

Returns to scale	2019	2020	%Change
Constant	48 (31.8%)	59 (39.1%)	23%
Decreasing	80 (53%)	62 (41.1%)	− 23%
Increasing	23 (15.2)	30 (19.9%)	30%

See Table 11 for Detailed scores for all health facilities

As per Table 10, the number of PCH that showed CRS increased from 13 to 35 facilities. There was also a reduction in PCH experiencing DRS and IRS. The number of SCH experiencing complete scale efficiency (CRS) increased from 1 to 2 health facilities in 2020. However, the number of SCH experiencing DRS reduced by 2, and those exhibiting IRS increased from 0 to 1 in 2020. The number of OLHF exhibiting CRS increased from 34 to 35, whiles those exhibiting DRS reduced from 22 to 14 and IRS increased from 20 to 27.

**Table 10:** Changes in health facilities exhibiting CRS, DRS and IRS; Source: Authors’ calculation based on field data

Facility category	CRS		DRS		IRS	
	2019	2020	2019	2020	2019	2020
*Type of facility*						
Primary Care	13	35	53	45	3	2
Secondary Care	1	2	5	3		1
Other Lower Level Fac	34	35	22	14	20	27

## Discussion

The study sought to examine the level and changes in efficiency from 2019 to 2020 as well as the determinants of variation in the efficiency of CHAG health facilities. The findings in general indicate that 59 health facilities (39%) out of a total of 151 in 2020 were efficient, with an average TE score of 0.73, compared to 48 health facilities (32%) with an average TE score of 0.67 that were efficient in 2019. The results and their implications for further research and policy are discussed below.

The results indicate that there was a net increase of 26% in inputs, driven mostly by a major increase (131%) in temporary non-clinical staff, and attrition of about 40% and 54% of temporary clinical staff and permanent non-clinical staff respectively. On the contrary, there was a net reduction of 34% in outputs, driven mainly by a reduction in in-patient days (37%), immunization (11%), outpatients visits and laboratory test of 9% respectively. The reason for replacing permanent non-clinical staff with temporal non-clinical staff is not directly apparent. It may well be that the permanent non-clinical staff had transitioned through the normal process (retirement, transfers etc.) and were replaced with temporal hands to help deal with the pressure arising from the COVID-19 pandemic in 2020. Notwithstanding the above explanation, it is important to emphasise that an attrition of 54% of permanent staff in a single year and the need to fill the gap with 131% increase in temporal staff of the same category may also reflect the poor nature of human resource practices and planning among the sampled health facilities. The net reduction in 2020 output may be attributed to the effect of the COVID-19 pandemic. There is evidence to suggest that the COVID-19 pandemic reduced inpatient days and OPD attendance in health facilities even though the situation improved later on [27, 28].

It is however significant to know that inspite of the COVID-19 pandemic, average TE score and sample of health facilities operating at the frontier improved from the 2019 level by 10 and 22 percentage points respectively. More importantly, the average TE score (0.74) and sample of health facilities operating at the frontier (39%) in 2020 constituted an improvement on earlier findings on mission health facilities (i.e. a 2014 paper based on 2005 data and using the same inputs/output), where the average TE score was 0.69 with 21.4% of the sample found to operate at the frontier [12]. The improvements may be related to internal efforts within CHAG and its health facilities to improve care delivery and for that matter efficiency. CHAG for example, has been collaborating with an NGO (PharmAccess) to implement strategic interventions like; Claim-IT, Med4All and recently SafeCare, to respectively improve insurance claims management, pharmaceutical supply chain and quality healthcare delivery [29–31]. The aggregate effect of these strategic interventions as well as local efforts may together be responsible for the improvement in average efficiency and the percentage of CHAG health facilities that were found to be efficient in 2020 compared to 2019 and 2014.

Although the overall average TE, PTE and SE scores were relatively high, it is important to point out that it is not the result of a higher number of health facilities being efficient but rather a few health facilities with very high efficiency scores. For instance, the proportion of the sampled health facilities that were efficient from a fully technical (31.8% in 2019 and 39.2% in 2020), pure technical (34.4% in 2019 and 44.4% in 2020) and scale (49% in both years) efficiency perspective were low. This implies that only a small number of health facilities were efficient. This will mean the need for health facility managers to work to improve the levels of efficiency in their health facilities. An option for health facility managers will be to improve outputs in line with the estimates for the different outputs at the different hierarchy of health facility as per the estimates in Table 7, especially for those health facilities that are experiencing increasing returns to scale. However, for most of these health facilities, increasing output may mean efforts to stimulate demand, which may depend on the size of the catchment population and ability of patients to pay especially in the absence of insurance. Given the current national health insurance coverage (54% in 2021) [32], which is likely to be lower among rural dwellers where CHAG health facilities operate, efforts to stimulate demand may end up being counterproductive, especially if the new users are not able to pay for their care.

In the absence of a bigger catchment population and higher health insurance coverage to contribute to the extra demand and also pay for services rendered, health facility managers and policy makers at CHAG can work together to consider the option of input redistribution as suggested by Jehu-Appiah et al. [12]. A typical case is PCH that experienced the lowest average efficiency score. For example, PCH exhibiting CRS had average clinical (153) and non-clinical [27] staff that were lower than average clinical (163) and non-clinical (49) staff for PCH exhibiting DRS. Even more importantly, PCH experiencing DRS had relatively smaller number of beds (88) compared to their counterparts experiencing CRS (106). This clearly indicate that PCH experiencing DRS are over staffed. Given that existing capacity will not support expansion in output in the short-term, redistribution of staff to under staffed PCH will be an option to pursue. In the current case, PCH experiencing IRS seem to be understaffed, with average clinical [20] and non-clinical [17] staff that is much lower than their CRS counterparts. Thus, PCH experiencing IRS could be candidates to receive excess staff from those experiencing DRS.The situation for OLHF seem not to be entirely different as the aggregate of clinical and non-clinical staff (41) for those experiencing CRS is lower than those experiencing DRS (64). In addition, the staff redistribution could also be from PCH to OLHF since PCH have a higher proportion of health facilities experiencing DRS wheras OLHF have a higher proportion of health facilities experiencing IRS.

It is important however, to emphasise that staff reallocation may not be easy to carry out especially if the direction of reallocation is from relatively bigger size and better resourced to smaller size health facilities located in resource-poor settings. There is vast literature on different cadre of health care staff refusing postings to resource-poor settings [33–35], resulting in over concentration of healthcare staff in well resourced health facilities in urban centres. Thus, without motivation and compelling incentives, a staff reallocation exercise is unlikely to succeed.

Consistent with the existing literature [11, 12], the findings equally indicate that bigger health facilities (SCH in this study) were more efficient than relatively smaller size health facilities (PCH and OLHF). In many LMICs such as Ghana, bigger hospitals are located in bigger towns and cities, making it possible for them to have access to different cadre of skilled health workforce [33–35] and thereby improving decision-making capacity. Additionally, their big size means that they can benefit from economies of scale in several areas of their operations. This suggest that bigger health facilities can take advantage of their decision-making capacity and size to be both technically and scale efficient compared to their relatively small counterparts.

Notwithstanding the uniqueness of the current study in terms of its access to a panel dataset of 151 facilities that covers both pre-and during COVID-19 periods, there are limitations that are worth noting. First, the study uses a sample of health facilities that are not homogeneous and therefore can result in variation in the quality of labour inputs. Also the health facilities were not adjusted for case-mix. This may have implications for changes either to inputs or whole units for purposes of improving efficiency. Finally, we acknowledge that output indcators such as Disability Adjusted-Life Expectancy (DALE) and Quality-Adjusted Life Years (QALY) better capture the objective function of health facilities [15]. However, given their unavailability, proxy indicators that have been used by prior studies [11–15] were used.

## Conclusion

The findings of the study indicate that overall average efficiency in CHAG health facilities has improved over the past two decades. Additionally, the fact that CHAG health facilities still improved their level of efficiency even in the midst of COVID-19 suggest the extent to which they have built their levels of resilience to external shocks such as COVID-19 over the years. The results are also in line with existing literature that indicates that bigger health facilities are relatively more efficient than their smaller counterparts.

Nevertheless, it is crucial to emphasise the need for health facility managers and decision-makers at CHAG to examine the factors that promoted efficiency improvements in those health facilities that were efficient so that less efficient health facilities can learn from them to improve on their levels of efficiency. Added to this is also the need to improve health insurance coverage since that will be crucial in ensuring effective additional demand for health services and therefore improvement in output, especially for those health facilities experiencing IRS and will therefore need to improve their outputs. For those experiencing DRS, staff reallocation has been suggested. Thus, decision makers, both in health facilities and CHAG will need to collaborate and carefully plan any staff reallocation exercise in a manner that meets the needs and aspirations of the different stakeholders.Strengthening the human resources planning function will also be important in managing the movement of the different cadre of health staff to avoid adverse incidence such as the 54% attrition of permanent staff in 2020. This will be important in limiting opposition to the reallocation exercise and therefore ensure success.

## Data Availability

All data generated and analysed during this study are included in this manuscript.

## Appendix 1: List of additional tables on efficiency computation

See Tables 11, 12 and 13.

**Table 11:** List of facilities with overall technical efficiency score of 100% and exhibiting CRS; Source: Based on field data for 2020

No	Name of facility
1	Anglican Clinic, Yelwoko
2	Baptist Medical Center—Nalerigu
3	Calvary Charismatic Baptist Medical Centre, Atwima Mim
4	Catholic Clinic, Barchabordo
5	Catholic Clinic—Phc Salaga
6	Catholic Hospital, Battor
7	Central Charismatic Baptist Medical Centre, Gyinyase
8	Dabaa Hope Hospital, Dabaa
9	E.P. Church Clinic, Wapuli
10	Evangelical Church Of Ghana Hospital, Kpandai
11	Emmanuel Eye Medical Centre
12	Faith Evangelical Mission Hospital
13	Fame Clinic, Tatindo
14	Father Thomas Alan Rooney Memorial Hospital
15	Holy Child Catholic Hospital, Fijai
16	Holy Spirit Clinic, Dantano
17	Manna Mission Hospital
18	Mercy Women's Catholic Hospital
19	Methodist Medical Centre, Brodekwano
20	Methodist Medical Centre, Dagyamen
21	Methodist Medical Centre, Kwakuanya
22	Pentecost Hospital, Madina
23	Pope Francis Health Centre, Komfourkrom
24	Powerhouse Hospital
25	Presbyterian Health Centre, Assin Nsuta
26	Presbyterian Health Centre, Kwamesua
27	Presbyterian Health Centre, Kyeremasu
28	Presbyterian Health Centre, Sumanduri
29	Presbyterian Regional Eye Centre, Yorogo
30	Presbyterian Phc, Loloto
31	Presbyterian Health Centre, Langbinsi
32	Presbyterian Chps, Amonie
33	Presbyterian Health Centre, Enchi
34	Presbyterian Health Centre, Jankufa
35	Presbyterian Health Centre, Kwadwokumikrom
36	Queen Of Peace Clinic, Sabuli
37	Richard Novati Catholic Hospital
38	Salvation Army Health Centre—Adaklu—Sofa
39	Salvation Army Health Centre, Ajumako Ochiso
40	Saviour Community Hospital, Bonwire
41	Seventh Day Adventist Clinic- Dadieso
42	SDA Hospital, Obuasi
43	SDA Hospital, Tamale
44	SDA Hospital, Kwadaso-Kumasi
45	St. Mary's Hospital, Drobo
46	St. Alban's Clinic (The Refugee Camp)
47	St. Anthony's Hospital
48	St. Dominic Hospital, Akwatia
49	St. Elizabeth Hospital, Hwidiem
50	St. Joseph’s Clinic-Wenchi Koasi
51	St. Marks Anglican Clinic
52	St. Martin De Porres Hospital, Eikwe
53	St. Martins Memorial Hospital—Shukura
54	St. Matthew's Hospital, Ampenkro
55	St. Theresa's Hospital, Nandom
56	St. Stella's Clinic, Karni
57	Presbyterian Health Centre, Suma Ahenkro
58	Salvation Army Hospital, Wiamoase
59	Todah Hospital, Obuasi

**Table 12:** Health facilities requiring improvement in efficiency scores; Source: Based on field data for 2020

No	Name	PTE	SE	TE	RTS
1.	Adventist Hospital, Breman	0.71	0.73	0.52	Decreasing
2.	Akomaa Memorial SDA Hospital	0.41%	0.85	0.34	Decreasing
3.	Anglican Clinic, Bonzain	0.19%	0.94	0.18	Increasing
4.	Anglican Health Centre, Tano-Odumase	0.85%	1	0.85	Decreasing
5.	Bebu Methodist Clinic	0.37%	0.93	0.34	Increasing
6.	Bryant Mission Hospital	0.61%	0.77	0.47	Decreasing
7.	Catholic Clinic, Oku	0.88%	1	0.88	Decreasing
8.	Church Of Christ Mission Clinic, Yendi	0.72%	0.96	0.69	Increasing
9.	Church Of God Clinic, Ahwerewam	0.37%	0.99	0.36	Increasing
10.	Church Of God Clinic, Apaaso	0.11%	0.89	0.10	Increasing
11.	Church Of God Medical Centre, Banda-Nkwanta	0.79%	0.94	0.75	Increasing
12.	Fame Clinic—Benwoko	0.24%	0.92	0.22	Increasing
13.	Global Evangelical Mission Hospital Apromase	0.34%	0.98	0.33	Increasing
14.	Grace Spring Mission Hospital	0.66%	95%	0.63	Increasing
15.	Hart Adventist Hospital	0.58%	0.89	0.52	Decreasing
16.	Holy Family Hospital, Berekum	100%	0.71	0.71	Decreasing
17.	Holy Family Hospital, Nkawkaw	100%	0.85	0.85	Decreasing
18.	Holy Family Hospital, Techiman	100%	0.81	0.81	Decreasing
19.	Hopexchange Medical Centre	0.40%	0.68	0.27	Decreasing
20.	Immaculate Conception Health Centre, Kaleo	0.56%	1	0.56	Increasing
21.	Janie Speaks A.M.E Zion Hospital, Afrancho	0.42%	0.64	0.27	Decreasing
22.	Kom Presbyterian Clinic	0.35%	0.99	0.35	Increasing
23.	Livingspring Baptist Medical Centre-Atasomanso	0.7%	0.70	0.5	Increasing
24.	Margaret Marquart Catholic Hospital	0.100%	0.90	0.90	Decreasing
25.	Martyrs Of Uganda Health Centre, Bole	0.69%	0.99	0.68	Decreasing
26.	Mary Ekuba Ewoo SDA Clinic, Akwidaa	0.92%	0.88	0.81	Increasing
27.	Mater Ecclesiae Hospital, Sokode	0.38%	0.94	0.36	Decreasing
28.	Mathias Catholic Hospital, Yeji	100%	0.91	0.91	Decreasing
29.	Methodist Medical Center, Adum	0.48%	0.71	0.34	Increasing
30.	Methodist Medical Centre, Apagya	0.85%	0.84	0.71	Decreasing
31.	Our Lady Of Grace Hospital, Breman-Asikuma	0.92%	0.88	0.81	Decreasing
32.	Pentecost Clinic—Kasapin	0.53%	0.90	0.47	Decreasing
33.	Pentecost Hospital, Tarkwa	0.87%	0.89	0.78	Decreasing
34.	Ph Anglican Eye Clinic, Jachie	0.70%	0.88	0.62	Decreasing
35.	Pope John Paul Ii Medical Centre	0.46%	0.78	0.36	Decreasing
36.	Presbyterian Clinic-Antwirifo	100%	0.96	0.96	Increasing
37.	Presbyterian Health Centre, Papueso	0.88%	0.64	0.57	Increasing
38.	Presbyterian Health Centre, Widana	100%	0.92	0.92	Decreasing
39.	Presbyterian Hospital, Dormaa Ahenkro	0.92%	0.71	0.65	Decreasing
40.	Presbyterian Phc, Salaga	0.9%	0.98	0.9	Increasing
41.	Sacred Heart Catholic Hospital	0.99%	0.96	0.95	Decreasing
42.	SDA Clinic—Sefwi Amoaya	0.88	0.89	0.78	Increasing
43.	SDA Clinic—Wa	0.74	0.99	0.73	Decreasing
44.	SDA Clinic And Maternity, Sefwi Punikrom	0.37	0.98	0.36	Increasing
45.	SDA Clinic Denkyira Domimase	0.50	0.95	0.48	Decreasing
46.	SDA Hospital Asamang	0.52	0.80	0.42	Decreasing
47.	SDA Hospital, Dwinase	1	0.93	0.93	Decreasing
48.	SDA Hospital, Gbawe	0.41	0.76	0.31	Decreasing
49.	SDA Hospital, Koforidua	1	0.78	0.78	Decreasing
50.	SDA Hospital, Namong	0.66	0.77	0.51	Decreasing
51.	SDA Hospital, Sunyani	0.95	0.87	0.83	Decreasing
52.	SDA Hospital, Wiamoase	0.82	0.90	0.74	Decreasing
53.	St Peter's Hospital—Jacobu	0.52	0.81	0.42	Decreasing
54.	St. Andrew's Catholic Hospital, Kordiabe	0.36	0.96	0.35	Decreasing
55.	St. Anne's Hospital, Damongo	0.55	0.85	0.47	Decreasing
56.	St. Anne's Polyclinic	0.27	0.91	0.25	Decreasing
57.	St. Anthony's Clinic	0.42	0.97	0.41	Increasing
58.	St. Benito Menni Hospital, Dompoase-Adansi	0.83	0.92	0.77	Decreasing
59.	St. Christopher Health Centre, Dapouri	1	0.86	0.86	Increasing
60.	St. Dominic Clinic, Cherembo	0.67	0.99	0.66	Increasing
61.	St. Edward's Hospital, Dwinyama	0.55	940	0.51	Decreasing
62.	St. Francis Xavier Hospital	1	0.77	0.77	Decreasing
63.	St. Georges Clinic, Liati	0.35	1	0.34	Increasing
64.	St. Gregory Catholic Hospital, Gomoa Budumburam	1	0.86	0.86	Decreasing
65.	St. Ignatius Health Centre, Lassia Tuolu	0.77	0.88	0.68	Increasing
66.	St. James Clinic	0.94	1	0.94	Increasing
67.	St. John Health Centre, Akim Ofoase	0.38	0.86	0.33	Decreasing
68.	St. John Of God Hospital, Amrahia	0.53	0.97	0.51	Decreasing
69.	St. John Of God Hospital, Duayaw-Nkwanta	0.99	0.96	0.94	Decreasing
70.	St. John Of God Hospital, Sefwi-Asafo	1	0.61	0.61	Decreasing
71.	St. Joseph Health Centre, Nakolo	0.57	0.96	0.55	Increasing
72.	St. Joseph's Clinic, Bechem	0.3	0.41	1	Increasing
73.	St. Joseph's Hospital, Jirapa	0.77	0.69	0.54	Decreasing
74.	St. Joseph's Hospital, Koforidua	0.65	0.66	0.43	Decreasing
75.	St. Joseph's Hospital, Nkwanta, Oti	0.49	0.76	0.37	Decreasing
76.	St. Lucas Hospital, Wiaga	0.46	0.90	0.42	Decreasing
77.	St. Luke Catholic Hospital, Apam	0.95	0.79	0.75	Decreasing
78.	St. Luke's Clinic, Chinderi	0.45	0.89	0.40	Decreasing
79.	St. Martin Memorial Hospital—Dansoman	0.55	0.82	0.45	Decreasing
80.	St. Martin's Catholic Hospital, Agroyesum	0.73	0.87	0.64	Decreasing
81.	St. Martin's De Porres Health Centre, Eremon	0.80	1	0.79	Decreasing
82.	St. Martin's Memorial Hospital—Ashaiman	0.76	0.67	0.51	Decreasing
83.	St. Mary Theresa Hospital	1	0.85	0.85	Decreasing
84.	St. Michael's Hospital, Pramso	0.90	0.63	0.57	Decreasing
85.	St. Patrick's Hospital, Offinso	1	0.90	0.90	Decreasing
86.	St. Theresa's Hospital, Nkoranza	0.8	1	0.87	Increasing
87.	St. Vincent Depaul Clinic, Drobonso	0.56	0.97	0.54	Increasing
88.	Tanoah Baptist Medical Centre, Opuniase	0.4	0.94	0.40	Increasing
89.	Tatale District Hospital, Tatale	0.89	0.90	0.81	Decreasing
90.	True Faith Hospital—Kumawu Bodomase	0.25	1	0.25	Decreasing
91.	Valley View Adventist Hospital, Oyibi	0.35	0.83	0.29	Decreasing
92.	Valley View Adventist Hospital, Techiman	0.72	0.95	0.69	Decreasing

**Table 13:** Percentage improvement in outputs required for efficiency; Source: Based on field data for 2020

No.	Name	Inpatient	Outpatient	ANC	Immunizations	Deliveries	Lab tests
1.	Adventist Hospital, Breman	40%	40%	288%	1231%	374%	40%
2.	Akomaa Memorial SDA Hospital	146%	146%	513%	158%	217%	146%
3.	Anglican Clinic, Bonzain	101,868%	417%	417%	–	834%	417%
4.	Anglican Health Centre, Tano-Odumase	–	18%	18%	62,459%	19%	20%
5.	Bebu Methodist Clinic	–	427%	171%	171%	171%	238%
6.	Bryant Mission Hospital	111%	64%	81%	64%	97%	283%
7.	Catholic Clinic, Oku	–	149%	14%	56%	14%	14%
8.	Church Of Christ Mission Clinic, Yendi	39%	39%	322%	2937%	39%	39%
9.	Church Of God Clinic, Ahwerewam	431%	196%	174%	–	174%	174%
10.	Church Of God Clinic, Apaaso	–	805%	805%	–	805%	881%
11.	Church Of God Medical Centre, Banda-Nkwanta	–	26%	–	–	–	26%
12.	Fame Clinic—Benwoko	–	322%	322%	322%	1657%	322%
13.	Global Evangelical Mission Hospital Apromase	195%	195%	215%	667%	381%	702%
14.	Grace Spring Mission Hospital	93%	51%	51%	729%	51%	51%
15.	Hart Adventist Hospital	71%	71%	71%	71%	136%	87%
16.	Holy Family Hospital, Berekum	0%	0%	0%	0%	0%	0%
17.	Holy Family Hospital, Nkawkaw	0%	0%	0%	0%	0%	0%
18.	Holy Family Hospital, Techiman	0%	0%	0%	0%	0%	0%
19.	Hopexchange Medical Centre	245%	150%	906%	298%	2905%	150%
20.	Immaculate Conception Health Centre, Kaleo	–	78%	78%	78%	78%	78%
21.	Janie Speaks A.M.E Zion Hospital, Afrancho	352%	159%	138%	138%	138%	138%
22.	Kom Presbyterian Clinic	–	96,040%	187%	187%	407%	187%
23.	Livingspring Baptist Medical Centre-Atasomanso	1243%	1243%	2377%	–	1243%	1414%
24.	Margaret Marquart Catholic Hospital	0%	0%	0%	0%	0%	0%
25.	Martyrs Of Uganda Health Centre, Bole	45%	45%	100%	45%	45%	81%
26.	Mary Ekuba Ewoo SDA Clinic, Akwidaa	–	95%	9%	–	9%	166%
27.	Mater Ecclesiae Hospital, Sokode	161%	161%	231%	161%	202%	691%
28.	Mathias Catholic Hospital, Yeji	0%	0%	0%	–	0%	0%
29.	Methodist Medical Center, Adum	–	109%	–	–	–	761%
30.	Methodist Medical Centre, Apagya	46,498%	18%	138%	18%	18%	18%
31.	Our Lady Of Grace Hospital, Breman-Asikuma	9%	15%	9%	33%	23%	83%
32.	Pentecost Clinic—Kasapin	–	263%	90%	90%	90%	90%
33.	Pentecost Hospital, Tarkwa	15%	15%	257%	370%	204%	15%
34.	Ph Anglican Eye Clinic, Jachie	–	43%	–	–	–	133%
35.	Pope John Paul Ii Medical Centre	116%	116%	141%	116%	168%	220%
36.	Presbyterian Clinic-Antwirifo	–	0%	–	–	–	0%
37.	Presbyterian Health Centre, Papueso	–	222%	13%	13%	13%	13%
38.	Presbyterian Health Centre, Widana	0%	0%	0%	0%	0%	0%
39.	Presbyterian Hospital, Dormaa Ahenkro	43%	9%	9%	738%	9%	285%
40.	Presbyterian Phc, Salaga	986%	986%	–	–	–	986%
41.	Sacred Heart Catholic Hospital	1%	24%	1%	1%	1%	1%
42.	SDA Clinic—Sefwi Amoaya	14%	25%	14%	–	309%	14%
43.	SDA Clinic—Wa	35%	35%	483%	–	574%	35%
44.	SDA Clinic And Maternity, Sefwi Punikrom	174%	174%	174%	–	174%	174%
45.	SDA Clinic Denkyira Domimase	–	159%	99%	99%	99%	99%
46.	SDA Hospital Asamang	95%	91%	191%	109%	194%	194%
47.	SDA Hospital, Dwinase	0%	0%	0%	0%	0%	0%
48.	SDA Hospital, Gbawe	146%	146%	256%	282%	395%	262%
49.	SDA Hospital, Koforidua	0%	0%	0%	0%	0%	0%
50.	SDA Hospital, Namong	50%	50%	217%	50%	132%	50%
51.	SDA Hospital, Sunyani	5%	5%	165%	5%	7%	5%
52.	SDA Hospital, Wiamoase	22%	22%	22%	22%	22%	1018%
53.	St Peter's Hospital—Jacobu	92%	108%	92%	3242%	191%	144%
54.	St. Andrew's Catholic Hospital, Kordiabe	175%	175%	378%	175%	175%	175%
55.	St. Anne's Hospital, Damongo	82%	95%	82%	82%	82%	120%
56.	St. Anne's Polyclinic	265%	265%	265%	265%	265%	335%
57.	St. Anthony's Clinic	–	161%	138%	138%	147%	138%
58.	St. Benito Menni Hospital, Dompoase-Adansi	20%	20%	70%	20%	20%	84%
59.	St. Christopher Health Centre, Dapouri	–	0%	0%	0%	0%	0%
60.	St. Dominic Clinic, Cherembo	197%	1422%	–	–	50%	667%
61.	St. Edward's Hospital, Dwinyama	82%	82%	86%	82%	109%	82%
62.	St. Francis Xavier Hospital	0%	0%	0%	0%	0%	0%
63.	St. Georges Clinic, Liati	–	189%	189%	189%	276%	189%
64.	St. Gregory Catholic Hospital, Gomoa Budumburam	0%	0%	0%	0%	0%	0%
65.	St. Ignatius Health Centre, Lassia Tuolu	30%	30%	30%	136%	30%	30%
66.	St. James Clinic	–	7%	7%	7%	276%	117%
67.	St. John Health Centre, Akim Ofoase	–	161%	736%	161%	161%	161%
68.	St. John Of God Hospital, Amrahia	95%	90%	138%	90%	90%	90%
69.	St. John Of God Hospital, Duayaw-Nkwanta	14%	16%	35%	17%	98%	2%
70.	St. John Of God Hospital, Sefwi-Asafo	0%	0%	0%	0%	0%	0%
71.	St. Joseph Health Centre, Nakolo	1916%	76%	76%	1690%	76%	76%
72.	St. Joseph's Clinic, Bechem	–	2860%	–	–	–	–
73.	St. Joseph's Hospital, Jirapa	29%	29%	33%	29%	29%	29%
74.	St. Joseph's Hospital, Koforidua	56%	55%	292%	566%	696%	142%
75.	St. Joseph's Hospital, Nkwanta, Oti	104%	104%	871%	136%	104%	104%
76.	St. Lucas Hospital, Wiaga	424%	117%	253%	1748%	117%	252%
77.	St. Luke Catholic Hospital, Apam	5%	40%	45%	39%	5%	5%
78.	St. Luke's Clinic, Chinderi	121%	121%	270%	121%	121%	121%
79.	St. Martin Memorial Hospital—Dansoman	130%	80%	327%	5128%	80%	80%
80.	St. Martin's Catholic Hospital, Agroyesum	36%	58%	36%	683%	36%	36%
81.	St. Martin's De Porres Health Centre, Eremon	–	25%	25%	25%	25%	25%
82.	St. Martin's Memorial Hospital—Ashaiman	31%	31%	96%	2855%	153%	137%
83.	St. Mary Theresa Hospital	0%	0%	0%	0%	0%	0%
84.	St. Michael's Hospital, Pramso	48%	11%	14%	4291%	26%	89%
85.	St. Patrick's Hospital, Offinso	0%	0%	0%	0%	0%	0%
86.	St. Theresa's Hospital, Nkoranza	15%	37%	25%	15%	15%	15%
87.	St. Vincent Depaul Clinic, Drobonso	79%	554%	79%	274%	79%	79%
88.	Tanoah Baptist Medical Centre, Opuniase	132%	132%	253%	–	132%	976%
89.	Tatale District Hospital, Tatale	12%	12%	49%	985%	12%	12%
90.	True Faith Hospital—Kumawu Bodomase	295%	513%	295%	295%	295%	2569%
91.	Valley View Adventist Hospital, Oyibi	182%	182%	344%	293%	642%	182%
92.	Valley View Adventist Hospital, Techiman	38%	38%	91%	420%	139%	38%

## Appendix 2: Traditional and biased corrected efficiency estimates

See Tables 14, 15, 16, and 17.

**Table 14:** Correlation between traditional and bias corrected efficiency scores

Model type	Correlation coefficeint (Kendall’s tau b)	p value
Full (2019 2020)	0.7970	0.00
2019 efficiency scores	0.7193	0.00
2020 efficiency scores	0.7605	0.00

Bias correction based on a bootstrap of 2000 replications

**Table 15:** Original and bias corrected efficiency scores for sampled hospitals (2019 and 2020 combined)

Facility (DMU) name	Original score	Bias corrected	95% Confidence interval	
			(Lower bound)	(Upper bound)
Adventist Hospital, Breman	0.51	0.44	0.39	0.50
Akomaa Memorial SDA Hospital	0.35	0.31	0.29	0.34
SDA Clinic—Sefwi Amoaya	0.26	0.22	0.19	0.25
Anglican Clinic, Bonzain	0.39	0.32	0.27	0.38
Anglican clinic, Yelwoko	1.00	0.75	0.58	0.99
PH Anglican Eye Clinic, Jachie	0.64	0.55	0.49	0.63
Anglican Health Centre, Tano-Odumase	0.32	0.27	0.24	0.32
Baptist Medical Center—Nalerigu	1.00	0.74	0.57	0.99
St. Benito Menni Hospital, Dompoase-Adansi	0.50	0.44	0.41	0.49
Bryant Mission Hospital	0.53	0.45	0.42	0.52
Calvary Charismatic Baptist Medical Centre, Atwima Mim	0.92	0.77	0.60	0.91
Catholic Clinic, Barchabordo	0.14	0.12	0.09	0.14
Catholic Clinic, Oku	0.39	0.33	0.29	0.39
Catholic Clinic—PHC Salaga	1.00	0.76	0.58	0.99
Catholic Hospital, Battor	1.00	0.80	0.69	0.99
Central Charismatic Baptist Medical Centre, Gyinyase	0.40	0.34	0.29	0.39
Church of Christ Mission Clinic, Yendi	0.59	0.50	0.45	0.58
Church of God Clinic, Ahwerewam	0.27	0.23	0.21	0.26
Church of God Clinic, Apaaso	0.04	0.03	0.03	0.04
Church of God Medical Centre, Banda-Nkwanta	0.68	0.59	0.52	0.67
Dabaa Hope Hospital, Dabaa	0.58	0.49	0.44	0.57
E.P. Church Clinic, Wapuli	1.00	0.77	0.63	0.98
Evangelical Church of Ghana Hospital, Kpandai	0.48	0.39	0.33	0.47
Emmanuel Eye Medical Centre	1.00	0.80	0.65	0.99
Faith Evangelical Mission Hospital	0.81	0.71	0.66	0.80
Fame Clinic, Tatindo	1.00	0.74	0.54	0.98
Fame Clinic—Benwoko	1.00	0.77	0.62	0.98
Father Thomas Alan Rooney Memorial Hospital	0.73	0.64	0.59	0.72
Global Evangelical Mission Hospital Apromase	0.17	0.15	0.14	0.16
Hart Adventist Hospital	0.40	0.34	0.29	0.40
Holy Child Catholic Hospital, Fijai	1.00	0.77	0.63	0.98
Holy Family Hospital, Techiman	1.00	0.80	0.66	0.98
Holy Family Hospital, Nkawkaw	1.00	0.84	0.74	0.98
Holy Family Hospital, Berekum	0.85	0.73	0.64	0.83
Holy Spirit Clinic, Dantano	1.00	0.74	0.54	0.98
HopeXchange Medical Centre	0.40	0.35	0.32	0.40
Immaculate Conception Health Centre, Kaleo	1.00	0.75	0.56	0.98
Janie Speaks A.M.E Zion Hospital, Afrancho	0.43	0.38	0.33	0.43
Kom Presbyterian Clinic	1.00	0.78	0.66	0.98
LivingSpring Baptist Medical Centre-Atasomanso	0.12	0.10	0.08	0.12
Manna Mission Hospital	0.96	0.83	0.73	0.94
Margaret Marquart Catholic Hospital	0.90	0.80	0.75	0.89
Martyrs Of Uganda Health Centre, Bole	1.00	0.77	0.63	0.98
Mary Ekuba Ewoo SDA Clinic, Akwidaa	0.34	0.28	0.24	0.33
Mater Ecclesiae Hospital, Sokode	0.55	0.45	0.38	0.54
Mathias Catholic Hospital, Yeji	1.00	0.79	0.66	0.99
Mercy Women's Catholic Hospital	1.00	0.83	0.71	0.98
Methodist Medical Center, Adum	1.00	0.75	0.55	0.98
Bebu Methodist Clinic	1.00	0.77	0.62	0.99
Methodist Medical Centre, Apagya	1.00	0.79	0.67	0.98
Methodist Medical Centre, Brodekwano	0.32	0.27	0.23	0.32
Methodist Medical Centre, Dagyamen	1.00	0.74	0.53	0.98
Methodist medical Centre, Kwakuanya	1.00	0.80	0.66	0.98
SDA Hospital, Namong	0.64	0.56	0.52	0.64
Our Lady of Grace Hospital, Breman-Asikuma	0.90	0.80	0.73	0.88
Pentecost Clinic—Kasapin	0.54	0.46	0.41	0.54
Pentecost Hospital, Madina	1.00	0.75	0.54	0.99
Pentecost Hospital, Tarkwa	1.00	0.78	0.65	0.99
Pope Francis Health Centre, Komfourkrom	1.00	0.76	0.59	0.98
Pope John Paul II Medical Centre	0.36	0.30	0.27	0.35
Powerhouse Hospital	0.20	0.17	0.14	0.20
Presbyterian Health Centre, Assin Nsuta	0.47	0.39	0.35	0.46
Presbyterian PHC, Salaga	1.00	0.77	0.63	0.98
Presbyterian Health Centre, Kwamesua	0.57	0.47	0.38	0.56
Presbyterian Health Centre, Kyeremasu	0.85	0.74	0.69	0.84
Presbyterian Health Centre, Sumanduri	1.00	0.84	0.73	0.98
Presbyterian Clinic-Antwirifo	0.29	0.24	0.20	0.28
Presbyterian Health Centre, Papueso	1.00	0.74	0.54	0.98
Presbyterian Regional Eye Centre, Yorogo	1.00	0.74	0.53	0.98
Presbyterian PHC, Loloto	1.00	0.75	0.55	0.98
Presbyterian Health Centre, Langbinsi	1.00	0.74	0.54	0.99
Presbyterian CHPS, Amonie	1.00	0.74	0.54	0.99
Presbyterian Health Centre, Enchi	1.00	0.83	0.68	0.99
Presbyterian Health Centre, Jankufa	0.56	0.47	0.37	0.55
Presbyterian Health Centre, Kwadwokumikrom	1.00	0.77	0.60	0.98
Presbyterian Hospital, Dormaa Ahenkro	1.00	0.76	0.59	0.99
Queen of Peace Clinic, Sabuli	1.00	0.75	0.59	0.98
Richard Novati Catholic Hospital	1.00	0.77	0.60	0.98
Sacred Heart Catholic Hospital	1.00	0.80	0.68	0.98
Salvation Army Health Centre—Adaklu—Sofa	0.37	0.31	0.28	0.36
Salvation Army Health Centre, Ajumako Ochiso	0.64	0.53	0.47	0.63
Saviour Community Hospital, Bonwire	0.23	0.20	0.18	0.23
SDA Clinic and Maternity, Sefwi Punikrom	0.32	0.27	0.25	0.31
SDA clinic Denkyira Domimase	0.87	0.74	0.66	0.85
Seventh Day Adventist Clinic- Dadieso	0.98	0.83	0.74	0.97
SDA Clinic—WA	0.62	0.52	0.44	0.61
SDA Hospital Asamang	0.66	0.56	0.50	0.65
SDA Hospital, Koforidua	0.93	0.82	0.75	0.92
SDA Hospital, Dwinase	0.89	0.76	0.63	0.88
SDA Hospital, Obuasi	0.75	0.64	0.58	0.74
SDA Hospital, Sunyani	1.00	0.80	0.69	0.98
SDA Hospital, Tamale	1.00	0.85	0.76	0.98
SDA Hospital, Wiamoase	0.44	0.38	0.35	0.43
Valley View Adventist Hospital, Oyibi	0.27	0.23	0.21	0.26
SDA Hospital, Kwadaso-Kumasi	1.00	0.76	0.60	0.99
SDA Hospital, Gbawe	0.30	0.26	0.24	0.30
Grace Spring Mission Hospital	0.48	0.42	0.38	0.47
St. Dominic Clinic, Cherembo	1.00	0.75	0.53	0.99
St. Edward's Hospital, Dwinyama	0.53	0.44	0.39	0.52
St. James Clinic	0.53	0.45	0.40	0.52
St. John Health Centre, Akim Ofoase	0.27	0.22	0.19	0.27
St. Joseph's Hospital, Koforidua	0.72	0.63	0.56	0.71
St. Lucas Hospital, Wiaga	0.78	0.66	0.58	0.77
St. Mary Theresa Hospital	0.50	0.44	0.41	0.50
St. Mary's Hospital, Drobo	0.96	0.84	0.76	0.94
St. Michael's Hospital, Pramso	0.97	0.83	0.75	0.95
St Peter's Hospital—Jacobu	0.41	0.35	0.32	0.40
St. Alban's Clinic (The Refugee Camp)	1.00	0.82	0.73	0.98
St. Andrew's Catholic Hospital, Kordiabe	0.42	0.35	0.32	0.42
St. Anne's Polyclinic	0.17	0.14	0.12	0.17
St. Anthony's Clinic	1.00	0.82	0.69	0.98
St. Anthony's Hospital	0.65	0.56	0.51	0.64
St. Dominic Hospital, Akwatia	1.00	0.84	0.74	0.99
St. Elizabeth Hospital, Hwidiem	1.00	0.83	0.73	0.99
St. Francis Xavier Hospital	1.00	0.82	0.69	0.98
St. Georges Clinic, Liati	0.22	0.19	0.17	0.22
St. Gregory Catholic Hospital, Gomoa Budumburam	1.00	0.80	0.71	0.99
St. Ignatius Health Centre, Lassia Tuolu	1.00	0.75	0.57	0.98
St. John of God Hospital, Sefwi-Asafo	1.00	0.84	0.75	0.99
St. John of God Hospital, Amrahia	0.29	0.25	0.23	0.29
St. John of God Hospital, Duayaw-Nkwanta	1.00	0.85	0.74	0.98
St. Joseph’s Clinic-Wenchi Koasi	0.97	0.83	0.75	0.96
St. Joseph Health centre, Nakolo	0.85	0.72	0.63	0.84
St. Joseph's Hospital, Nkwanta, Oti	0.51	0.44	0.41	0.50
St. Joseph's Clinic, Bechem	1.00	0.74	0.54	0.99
St. Luke's Clinic, Chinderi	0.33	0.28	0.25	0.33
St. Luke catholic hospital, apam	0.71	0.63	0.59	0.70
St. Marks Anglican Clinic	0.54	0.46	0.41	0.53
St. Martin de Porres Hospital, Eikwe	1.00	0.82	0.74	0.99
St. Martin Memorial Hospital—Dansoman	0.56	0.47	0.42	0.55
St. Martin's Catholic Hospital, Agroyesum	0.77	0.66	0.61	0.76
St. Martins Memorial Hospital—Shukura	1.00	0.75	0.58	0.99
St. Martin's Memorial Hospital—Ashaiman	0.60	0.52	0.47	0.59
St. Matthew's hospital, ampenkro	1.00	0.78	0.63	0.98
St. Patrick's Hospital, Offinso	1.00	0.78	0.66	0.98
St. Theresa's hospital, Nkoranza	0.85	0.74	0.67	0.84
St. Theresa's Hospital, Nandom	1.00	0.75	0.59	0.99
St. Vincent DePaul Clinic, Drobonso	0.35	0.29	0.25	0.34
St. Christopher Health Centre, Dapouri	1.00	0.74	0.53	0.99
St. Joseph's Hospital, Jirapa	1.00	0.82	0.69	0.98
St. Martin's De Porres Health Centre, Eremon	0.80	0.68	0.57	0.79
St. Stella's Clinic, Karni	1.00	0.75	0.54	0.98
Presbyterian Health Centre, Suma Ahenkro	1.00	0.74	0.56	0.98
Tanoah Baptist Medical Centre, Opuniase	0.28	0.24	0.21	0.28
Tatale district hospital, tatale	1.00	0.77	0.62	0.99
Salvation Army Hospital, Wiamoase	1.00	0.77	0.64	0.99
Todah Hospital, Obuasi	1.00	0.75	0.54	0.99
True Faith Hospital—Kumawu Bodomase	0.11	0.09	0.08	0.11
Valley View Adventist Hospital, Techiman	0.55	0.45	0.37	0.54
St. Anne's Hospital, Damongo	0.62	0.53	0.47	0.61
Presbyterian Health Centre, Widana	1.00	0.75	0.57	0.98
Adventist Hospital, Breman	0.54	0.48	0.44	0.53
Akomaa Memorial SDA Hospital	0.30	0.26	0.25	0.30
SDA Clinic—Sefwi Amoaya	0.31	0.27	0.23	0.31
Anglican Clinic, Bonzain	0.19	0.17	0.15	0.19
Anglican clinic, Yelwoko	0.90	0.76	0.62	0.88
PH Anglican Eye Clinic, Jachie	0.66	0.57	0.49	0.65
Anglican Health Centre, Tano-Odumase	0.38	0.32	0.27	0.37
Baptist Medical Center—Nalerigu	1.00	0.81	0.69	0.99
St. Benito Menni Hospital, Dompoase-Adansi	0.71	0.60	0.53	0.70
Bryant Mission Hospital	0.59	0.50	0.45	0.58
Calvary Charismatic Baptist Medical Centre, Atwima Mim	1.00	0.78	0.63	0.98
Catholic Clinic, Barchabordo	1.00	0.74	0.54	0.99
Catholic Clinic, Oku	0.87	0.73	0.65	0.85
Catholic Clinic—PHC Salaga	1.00	0.81	0.66	0.98
Catholic Hospital, Battor	1.00	0.83	0.74	0.99
Central Charismatic Baptist Medical Centre, Gyinyase	1.00	0.82	0.70	0.98
Church of Christ Mission Clinic, Yendi	0.57	0.47	0.41	0.56
Church of God Clinic, Ahwerewam	0.36	0.31	0.27	0.36
Church of God Clinic, Apaaso	0.10	0.09	0.07	0.10
Church of God Medical Centre, Banda-Nkwanta	0.72	0.62	0.56	0.71
Dabaa Hope Hospital, Dabaa	1.00	0.83	0.73	0.99
E.P. Church Clinic, Wapuli	1.00	0.75	0.58	0.99
Evangelical Church of Ghana Hospital, Kpandai	1.00	0.75	0.54	0.99
Emmanuel Eye Medical Centre	1.00	0.76	0.60	0.98
Faith Evangelical Mission Hospital	1.00	0.77	0.61	0.98
Fame Clinic, Tatindo	1.00	0.77	0.64	0.99
Fame Clinic—Benwoko	0.23	0.19	0.16	0.23
Father Thomas Alan Rooney Memorial Hospital	1.00	0.75	0.57	0.99
Global Evangelical Mission Hospital Apromase	0.29	0.24	0.20	0.28
Hart Adventist Hospital	0.40	0.34	0.30	0.39
Holy Child Catholic Hospital, Fijai	0.85	0.72	0.62	0.84
Holy Family Hospital, Techiman	1.00	0.79	0.68	0.99
Holy Family Hospital, Nkawkaw	0.97	0.81	0.69	0.95
Holy Family Hospital, Berekum	1.00	0.88	0.80	0.98
Holy Spirit Clinic, Dantano	1.00	0.74	0.53	0.98
HopeXchange Medical Centre	0.38	0.34	0.32	0.38
Immaculate Conception Health Centre, Kaleo	0.47	0.40	0.35	0.47
Janie Speaks A.M.E Zion Hospital, Afrancho	0.41	0.35	0.31	0.40
Kom Presbyterian Clinic	0.35	0.29	0.25	0.34
LivingSpring Baptist Medical Centre-Atasomanso	0.05	0.04	0.04	0.05
Manna Mission Hospital	1.00	0.82	0.71	0.98
Margaret Marquart Catholic Hospital	1.00	0.82	0.71	0.99
Martyrs Of Uganda Health Centre, Bole	0.63	0.55	0.49	0.62
Mary Ekuba Ewoo SDA Clinic, Akwidaa	0.72	0.61	0.51	0.71
Mater Ecclesiae Hospital, Sokode	0.34	0.29	0.26	0.34
Mathias Catholic Hospital, Yeji	1.00	0.82	0.69	0.98
Mercy Women's Catholic Hospital	1.00	0.82	0.71	0.98
Methodist Medical Center, Adum	0.26	0.22	0.18	0.26
Bebu Methodist Clinic	0.32	0.27	0.23	0.32
Methodist Medical Centre, Apagya	0.84	0.71	0.61	0.83
Methodist Medical Centre, Brodekwano	0.75	0.61	0.47	0.74
Methodist Medical Centre, Dagyamen	0.37	0.30	0.25	0.36
Methodist medical Centre, Kwakuanya	1.00	0.77	0.62	0.99
SDA Hospital, Namong	0.63	0.55	0.52	0.62
Our Lady of Grace Hospital, Breman-Asikuma	0.81	0.69	0.60	0.79
Pentecost Clinic—Kasapin	0.51	0.43	0.38	0.51
Pentecost Hospital, Madina	1.00	0.74	0.55	0.98
Pentecost Hospital, Tarkwa	0.75	0.66	0.61	0.73
Pope Francis Health Centre, Komfourkrom	0.93	0.77	0.60	0.92
Pope John Paul II Medical Centre	0.30	0.26	0.22	0.30
Powerhouse Hospital	1.00	0.78	0.63	0.99
Presbyterian Health Centre, Assin Nsuta	1.00	0.75	0.57	0.98
Presbyterian PHC, Salaga	0.07	0.06	0.05	0.07
Presbyterian Health Centre, Kwamesua	1.00	0.82	0.68	0.99
Presbyterian Health Centre, Kyeremasu	1.00	0.75	0.56	0.99
Presbyterian Health Centre, Sumanduri	1.00	0.74	0.55	0.98
Presbyterian Clinic-Antwirifo	0.43	0.35	0.29	0.42
Presbyterian Health Centre, Papueso	0.80	0.67	0.58	0.79
Presbyterian Regional Eye Centre, Yorogo	1.00	0.75	0.54	0.99
Presbyterian PHC, Loloto	0.96	0.83	0.70	0.95
Presbyterian Health Centre, Langbinsi	1.00	0.78	0.60	0.98
Presbyterian CHPS, Amonie	1.00	0.80	0.65	0.99
Presbyterian Health Centre, Enchi	0.98	0.87	0.77	0.97
Presbyterian Health Centre, Jankufa	1.00	0.74	0.54	0.98
Presbyterian Health Centre, Kwadwokumikrom	1.00	0.75	0.53	0.98
Presbyterian Hospital, Dormaa Ahenkro	0.88	0.74	0.65	0.86
Queen of Peace Clinic, Sabuli	1.00	0.84	0.72	0.99
Richard Novati Catholic Hospital	1.00	0.74	0.54	0.98
Sacred Heart Catholic Hospital	0.91	0.78	0.70	0.90
Salvation Army Health Centre—Adaklu—Sofa	1.00	0.74	0.54	0.99
Salvation Army Health Centre, Ajumako Ochiso	1.00	0.82	0.72	0.99
Saviour Community Hospital, Bonwire	0.47	0.39	0.34	0.46
SDA Clinic and Maternity, Sefwi Punikrom	0.29	0.25	0.23	0.29
SDA clinic Denkyira Domimase	0.49	0.42	0.38	0.48
Seventh Day Adventist Clinic- Dadieso	1.00	0.80	0.66	0.98
SDA Clinic—WA	0.64	0.56	0.51	0.63
SDA Hospital Asamang	0.49	0.42	0.38	0.48
SDA Hospital, Koforidua	0.90	0.79	0.72	0.89
SDA Hospital, Dwinase	0.98	0.83	0.74	0.97
SDA Hospital, Obuasi	1.00	0.83	0.74	0.98
SDA Hospital, Sunyani	0.91	0.80	0.74	0.90
SDA Hospital, Tamale	1.00	0.76	0.60	0.98
SDA Hospital, Wiamoase	0.73	0.61	0.54	0.71
Valley View Adventist Hospital, Oyibi	0.23	0.20	0.18	0.23
SDA Hospital, Kwadaso-Kumasi	1.00	0.80	0.63	0.99
SDA Hospital, Gbawe	0.33	0.29	0.27	0.32
Grace Spring Mission Hospital	0.64	0.56	0.52	0.63
St. Dominic Clinic, Cherembo	0.66	0.55	0.42	0.65
St. Edward's Hospital, Dwinyama	0.48	0.41	0.38	0.47
St. James Clinic	0.79	0.68	0.60	0.78
St. John Health Centre, Akim Ofoase	0.38	0.32	0.28	0.37
St. Joseph's Hospital, Koforidua	0.56	0.48	0.43	0.55
St. Lucas Hospital, Wiaga	0.46	0.40	0.36	0.45
St. Mary Theresa Hospital	0.66	0.57	0.52	0.65
St. Mary's Hospital, Drobo	1.00	0.75	0.55	0.98
St. Michael's Hospital, Pramso	0.86	0.76	0.69	0.85
St Peter's Hospital—Jacobu	0.33	0.28	0.25	0.32
St. Alban's Clinic (The Refugee Camp)	1.00	0.82	0.68	0.98
St. Andrew's Catholic Hospital, Kordiabe	0.32	0.27	0.24	0.31
St. Anne's Polyclinic	0.18	0.15	0.13	0.18
St. Anthony's Clinic	0.42	0.34	0.28	0.41
St. Anthony's Hospital	0.71	0.58	0.48	0.70
St. Dominic Hospital, Akwatia	1.00	0.79	0.66	0.98
St. Elizabeth Hospital, Hwidiem	1.00	0.76	0.60	0.98
St. Francis Xavier Hospital	1.00	0.83	0.75	0.98
St. Georges Clinic, Liati	0.33	0.27	0.23	0.33
St. Gregory Catholic Hospital, Gomoa Budumburam	1.00	0.84	0.73	0.98
St. Ignatius Health Centre, Lassia Tuolu	0.66	0.55	0.49	0.65
St. John of God Hospital, Sefwi-Asafo	1.00	0.86	0.77	0.98
St. John of God Hospital, Amrahia	0.53	0.44	0.38	0.52
St. John of God Hospital, Duayaw-Nkwanta	0.98	0.83	0.71	0.97
St. Joseph’s Clinic-Wenchi Koasi	1.00	0.77	0.62	0.99
St. Joseph Health centre, Nakolo	0.52	0.46	0.42	0.51
St. Joseph's Hospital, Nkwanta, Oti	0.39	0.35	0.32	0.39
St. Joseph's Clinic, Bechem	0.02	0.01	0.01	0.02
St. Luke's Clinic, Chinderi	0.36	0.30	0.27	0.35
St. Luke catholic hospital, apam	0.92	0.80	0.73	0.91
St. Marks Anglican Clinic	1.00	0.75	0.54	0.99
St. Martin de Porres Hospital, Eikwe	1.00	0.80	0.70	0.98
St. Martin Memorial Hospital—Dansoman	0.52	0.46	0.43	0.51
St. Martin's Catholic Hospital, Agroyesum	0.69	0.59	0.52	0.68
St. Martins Memorial Hospital—Shukura	1.00	0.74	0.54	0.98
St. Martin's Memorial Hospital—Ashaiman	0.55	0.50	0.47	0.54
St. Matthew's hospital, ampenkro	1.00	0.84	0.75	0.98
St. Patrick's Hospital, Offinso	1.00	0.82	0.72	0.98
St. Theresa's hospital, Nkoranza	0.79	0.68	0.58	0.78
St. Theresa's Hospital, Nandom	1.00	0.76	0.59	0.98
St. Vincent DePaul Clinic, Drobonso	0.52	0.43	0.37	0.51
St. Christopher Health Centre, Dapouri	1.00	0.76	0.59	0.98
St. Joseph's Hospital, Jirapa	0.70	0.60	0.55	0.69
St. Martin's De Porres Health Centre, Eremon	0.77	0.66	0.59	0.76
St. Stella's Clinic, Karni	1.00	0.75	0.56	0.98
Presbyterian Health Centre, Suma Ahenkro	1.00	0.78	0.59	0.99
Tanoah Baptist Medical Centre, Opuniase	0.19	0.16	0.13	0.19
Tatale district hospital, tatale	0.61	0.53	0.49	0.60
Salvation Army Hospital, Wiamoase	1.00	0.81	0.72	0.99
Todah Hospital, Obuasi	1.00	0.75	0.57	0.98
True Faith Hospital—Kumawu Bodomase	0.25	0.21	0.17	0.25
Valley View Adventist Hospital, Techiman	0.56	0.47	0.42	0.55
St. Anne's Hospital, Damongo	0.53	0.46	0.43	0.52
Presbyterian Health Centre, Widana	1.00	0.79	0.67	0.98

**Table 16:** Original and bias corrected efficiency scores for sampled hospitals (2019 efficiency scores)

Facility (DMU) name	Original score	Bias corrected	95% Confidence interval	
			(Lower bound)	(Upper bound)
Adventist Hospital, Breman	0.61	0.53	0.48	0.60
Akomaa Memorial SDA Hospital	0.42	0.38	0.35	0.42
SDA Clinic—Sefwi Amoaya	0.26	0.23	0.20	0.25
Anglican Clinic, Bonzain	0.87	0.77	0.62	0.87
Anglican clinic, Yelwoko	1.00	0.78	0.59	0.99
PH Anglican Eye Clinic, Jachie	0.86	0.76	0.67	0.85
Anglican Health Centre, Tano-Odumase	0.40	0.35	0.31	0.40
Baptist Medical Center—Nalerigu	1.00	0.78	0.56	0.99
St. Benito Menni Hospital, Dompoase-Adansi	0.58	0.52	0.47	0.57
Bryant Mission Hospital	0.54	0.47	0.42	0.54
Calvary Charismatic Baptist Medical Centre, Atwima Mim	0.92	0.80	0.63	0.91
Catholic Clinic, Barchabordo	0.14	0.12	0.10	0.14
Catholic Clinic, Oku	0.39	0.34	0.30	0.39
Catholic Clinic—PHC Salaga	1.00	0.79	0.60	0.99
Catholic Hospital, Battor	1.00	0.83	0.71	0.99
Central Charismatic Baptist Medical Centre, Gyinyase	0.40	0.35	0.31	0.39
Church of Christ Mission Clinic, Yendi	0.66	0.57	0.48	0.65
Church of God Clinic, Ahwerewam	0.31	0.27	0.23	0.31
Church of God Clinic, Apaaso	0.04	0.03	0.03	0.04
Church of God Medical Centre, Banda-Nkwanta	0.92	0.80	0.69	0.91
Dabaa Hope Hospital, Dabaa	0.96	0.85	0.76	0.95
E.P. Church Clinic, Wapuli	1.00	0.80	0.64	0.99
Evangelical Church of Ghana Hospital, Kpandai	0.48	0.41	0.34	0.47
Emmanuel Eye Medical Centre	1.00	0.79	0.62	0.99
Faith Evangelical Mission Hospital	0.90	0.80	0.72	0.89
Fame Clinic, Tatindo	1.00	0.78	0.56	0.99
Fame Clinic—Benwoko	1.00	0.79	0.61	0.99
Father Thomas Alan Rooney Memorial Hospital	0.76	0.67	0.60	0.75
Global Evangelical Mission Hospital Apromase	0.18	0.16	0.15	0.18
Hart Adventist Hospital	0.51	0.44	0.38	0.50
Holy Child Catholic Hospital, Fijai	1.00	0.78	0.59	0.99
Holy Family Hospital, Techiman	1.00	0.81	0.66	0.99
Holy Family Hospital, Nkawkaw	1.00	0.85	0.73	0.99
Holy Family Hospital, Berekum	0.86	0.75	0.64	0.85
Holy Spirit Clinic, Dantano	1.00	0.78	0.56	0.99
HopeXchange Medical Centre	0.49	0.44	0.41	0.48
Immaculate Conception Health Centre, Kaleo	1.00	0.77	0.57	0.98
Janie Speaks A.M.E Zion Hospital, Afrancho	0.57	0.50	0.44	0.56
Kom Presbyterian Clinic	1.00	0.81	0.68	0.99
LivingSpring Baptist Medical Centre-Atasomanso	0.12	0.11	0.09	0.12
Manna Mission Hospital	1.00	0.82	0.70	0.99
Margaret Marquart Catholic Hospital	0.94	0.83	0.75	0.92
Martyrs Of Uganda Health Centre, Bole	1.00	0.80	0.64	0.99
Mary Ekuba Ewoo SDA Clinic, Akwidaa	0.34	0.29	0.25	0.34
Mater Ecclesiae Hospital, Sokode	0.78	0.67	0.57	0.77
Mathias Catholic Hospital, Yeji	1.00	0.81	0.68	0.99
Mercy Women's Catholic Hospital	1.00	0.86	0.73	0.98
Methodist Medical Center, Adum	1.00	0.78	0.56	0.99
Bebu Methodist Clinic	1.00	0.78	0.56	0.99
Methodist Medical Centre, Apagya	1.00	0.81	0.68	0.99
Methodist Medical Centre, Brodekwano	0.33	0.29	0.25	0.33
Methodist Medical Centre, Dagyamen	1.00	0.78	0.56	0.99
Methodist medical Centre, Kwakuanya	1.00	0.80	0.65	0.99
SDA Hospital, Namong	0.73	0.65	0.60	0.72
Our Lady of Grace Hospital, Breman-Asikuma	0.91	0.81	0.73	0.89
Pentecost Clinic—Kasapin	0.63	0.55	0.47	0.63
Pentecost Hospital, Madina	1.00	0.79	0.56	0.99
Pentecost Hospital, Tarkwa	1.00	0.79	0.64	0.99
Pope Francis Health Centre, Komfourkrom	1.00	0.78	0.60	0.99
Pope John Paul II Medical Centre	0.60	0.52	0.46	0.59
Powerhouse Hospital	0.31	0.26	0.21	0.30
Presbyterian Health Centre, Assin Nsuta	0.63	0.56	0.50	0.63
Presbyterian PHC, Salaga	1.00	0.80	0.64	0.99
Presbyterian Health Centre, Kwamesua	1.00	0.82	0.71	0.99
Presbyterian Health Centre, Kyeremasu	0.99	0.87	0.79	0.98
Presbyterian Health Centre, Sumanduri	1.00	0.86	0.76	0.99
Presbyterian Clinic-Antwirifo	0.29	0.25	0.21	0.28
Presbyterian Health Centre, Papueso	1.00	0.78	0.55	0.99
Presbyterian Regional Eye Centre, Yorogo	1.00	0.78	0.55	0.99
Presbyterian PHC, Loloto	1.00	0.78	0.56	0.99
Presbyterian Health Centre, Langbinsi	1.00	0.78	0.56	0.99
Presbyterian CHPS, Amonie	1.00	0.77	0.55	0.99
Presbyterian Health Centre, Enchi	1.00	0.86	0.73	0.99
Presbyterian Health Centre, Jankufa	1.00	0.78	0.58	0.99
Presbyterian Health Centre, Kwadwokumikrom	1.00	0.78	0.59	0.99
Presbyterian Hospital, Dormaa Ahenkro	1.00	0.78	0.59	0.99
Queen of Peace Clinic, Sabuli	1.00	0.78	0.61	0.99
Richard Novati Catholic Hospital	1.00	0.78	0.59	0.99
Sacred Heart Catholic Hospital	1.00	0.83	0.70	0.99
Salvation Army Health Centre—Adaklu—Sofa	0.59	0.51	0.45	0.59
Salvation Army Health Centre, Ajumako Ochiso	0.98	0.86	0.76	0.97
Saviour Community Hospital, Bonwire	0.30	0.26	0.23	0.29
SDA Clinic and Maternity, Sefwi Punikrom	0.40	0.34	0.30	0.39
SDA clinic Denkyira Domimase	1.00	0.82	0.70	0.99
Seventh Day Adventist Clinic- Dadieso	1.00	0.81	0.68	0.99
SDA Clinic—WA	0.62	0.54	0.47	0.61
SDA Hospital Asamang	0.68	0.60	0.52	0.67
SDA Hospital, Koforidua	1.00	0.89	0.80	0.99
SDA Hospital, Dwinase	0.89	0.78	0.65	0.88
SDA Hospital, Obuasi	0.97	0.86	0.77	0.96
SDA Hospital, Sunyani	1.00	0.81	0.68	0.99
SDA Hospital, Tamale	1.00	0.82	0.68	0.99
SDA Hospital, Wiamoase	0.47	0.41	0.36	0.46
Valley View Adventist Hospital, Oyibi	0.39	0.36	0.34	0.39
SDA Hospital, Kwadaso-Kumasi	1.00	0.78	0.56	0.99
SDA Hospital, Gbawe	0.44	0.40	0.37	0.44
Grace Spring Mission Hospital	0.59	0.52	0.46	0.59
St. Dominic Clinic, Cherembo	1.00	0.78	0.55	0.99
St. Edward's Hospital, Dwinyama	0.61	0.54	0.48	0.61
St. James Clinic	0.61	0.53	0.46	0.61
St. John Health Centre, Akim Ofoase	0.46	0.40	0.34	0.45
St. Joseph's Hospital, Koforidua	0.72	0.63	0.54	0.72
St. Lucas Hospital, Wiaga	0.98	0.87	0.78	0.97
St. Mary Theresa Hospital	0.53	0.47	0.43	0.53
St. Mary's Hospital, Drobo	1.00	0.83	0.72	0.99
St. Michael's Hospital, Pramso	1.00	0.83	0.72	0.99
St Peter's Hospital—Jacobu	0.45	0.39	0.36	0.44
St. Alban's Clinic (The Refugee Camp)	1.00	0.81	0.70	0.99
St. Andrew's Catholic Hospital, Kordiabe	0.62	0.55	0.50	0.61
St. Anne's Polyclinic	0.32	0.29	0.26	0.32
St. Anthony's Clinic	1.00	0.82	0.66	0.99
St. Anthony's Hospital	0.66	0.58	0.51	0.66
St. Dominic Hospital, Akwatia	1.00	0.84	0.72	0.99
St. Elizabeth Hospital, Hwidiem	1.00	0.82	0.68	0.99
St. Francis Xavier Hospital	1.00	0.85	0.71	0.99
St. Georges Clinic, Liati	0.30	0.26	0.23	0.30
St. Gregory Catholic Hospital, Gomoa Budumburam	1.00	0.80	0.64	0.99
St. Ignatius Health Centre, Lassia Tuolu	1.00	0.78	0.57	0.99
St. John of God Hospital, Sefwi-Asafo	1.00	0.83	0.72	0.99
St. John of God Hospital, Amrahia	0.32	0.28	0.25	0.32
St. John of God Hospital, Duayaw-Nkwanta	1.00	0.85	0.73	0.99
St. Joseph’s Clinic-Wenchi Koasi	1.00	0.83	0.72	0.99
St. Joseph Health centre, Nakolo	0.85	0.74	0.66	0.84
St. Joseph's Hospital, Nkwanta, Oti	0.53	0.46	0.42	0.52
St. Joseph's Clinic, Bechem	1.00	0.78	0.56	0.99
St. Luke's Clinic, Chinderi	0.52	0.46	0.41	0.51
St. Luke catholic hospital, apam	0.80	0.71	0.64	0.79
St. Marks Anglican Clinic	0.67	0.59	0.51	0.67
St. Martin de Porres Hospital, Eikwe	1.00	0.82	0.72	0.99
St. Martin Memorial Hospital—Dansoman	0.70	0.62	0.54	0.69
St. Martin's Catholic Hospital, Agroyesum	0.95	0.85	0.78	0.94
St. Martins Memorial Hospital—Shukura	1.00	0.79	0.59	0.99
St. Martin's Memorial Hospital—Ashaiman	0.65	0.57	0.52	0.64
St. Matthew's hospital, ampenkro	1.00	0.81	0.65	0.99
St. Patrick's Hospital, Offinso	1.00	0.79	0.63	0.99
St. Theresa's hospital, Nkoranza	0.86	0.75	0.66	0.85
St. Theresa's Hospital, Nandom	1.00	0.79	0.60	0.99
St. Vincent DePaul Clinic, Drobonso	0.48	0.41	0.34	0.47
St. Christopher Health Centre, Dapouri	1.00	0.78	0.57	0.99
St. Joseph's Hospital, Jirapa	1.00	0.85	0.72	0.99
St. Martin's De Porres Health Centre, Eremon	0.80	0.70	0.60	0.79
St. Stella's Clinic, Karni	1.00	0.78	0.55	0.99
Presbyterian Health Centre, Suma Ahenkro	1.00	0.78	0.55	0.99
Tanoah Baptist Medical Centre, Opuniase	0.28	0.25	0.21	0.28
Tatale district hospital, tatale	1.00	0.79	0.63	0.99
Salvation Army Hospital, Wiamoase	1.00	0.78	0.60	0.99
Todah Hospital, Obuasi	1.00	0.78	0.56	0.99
True Faith Hospital—Kumawu Bodomase	0.22	0.19	0.16	0.21
Valley View Adventist Hospital, Techiman	0.55	0.47	0.38	0.54
St. Anne's Hospital, Damongo	0.62	0.54	0.48	0.61
Presbyterian Health Centre, Widana	1.00	0.78	0.57	0.99

**Table 17:** Original and bias corrected efficiency scores for sampled hospitals (2020 efficiency scores)

Facility (DMU) name	Original score	Bias corrected	95% Confidence interval	
			(Lower bound)	(Upper bound)
Adventist Hospital, Breman	0.71	0.64	0.60	0.70
Akomaa Memorial SDA Hospital	0.41	0.37	0.34	0.40
SDA Clinic—Sefwi Amoaya	0.88	0.75	0.61	0.87
Anglican Clinic, Bonzain	0.19	0.17	0.15	0.19
Anglican clinic, Yelwoko	1.00	0.82	0.66	0.98
PH Anglican Eye Clinic, Jachie	0.70	0.60	0.50	0.69
Anglican Health Centre, Tano-Odumase	0.85	0.71	0.60	0.83
Baptist Medical Center—Nalerigu	1.00	0.79	0.64	0.99
St. Benito Menni Hospital, Dompoase-Adansi	0.83	0.73	0.66	0.83
Bryant Mission Hospital	0.63	0.54	0.48	0.63
Calvary Charismatic Baptist Medical Centre, Atwima Mim	1.00	0.79	0.61	0.99
Catholic Clinic, Barchabordo	1.00	0.77	0.53	0.99
Catholic Clinic, Oku	0.88	0.75	0.65	0.87
Catholic Clinic—PHC Salaga	1.00	0.78	0.57	0.99
Catholic Hospital, Battor	1.00	0.81	0.66	0.99
Central Charismatic Baptist Medical Centre, Gyinyase	1.00	0.78	0.53	0.98
Church of Christ Mission Clinic, Yendi	0.72	0.61	0.51	0.71
Church of God Clinic, Ahwerewam	0.37	0.31	0.27	0.36
Church of God Clinic, Apaaso	0.11	0.09	0.08	0.11
Church of God Medical Centre, Banda-Nkwanta	0.79	0.69	0.62	0.79
Dabaa Hope Hospital, Dabaa	1.00	0.83	0.72	0.99
E.P. Church Clinic, Wapuli	1.00	0.77	0.56	0.98
Evangelical Church of Ghana Hospital, Kpandai	1.00	0.78	0.55	0.99
Emmanuel Eye Medical Centre	1.00	0.77	0.53	0.99
Faith Evangelical Mission Hospital	1.00	0.80	0.62	0.99
Fame Clinic, Tatindo	1.00	0.78	0.60	0.99
Fame Clinic—Benwoko	0.24	0.20	0.16	0.23
Father Thomas Alan Rooney Memorial Hospital	1.00	0.78	0.59	0.99
Global Evangelical Mission Hospital Apromase	0.34	0.29	0.24	0.33
Hart Adventist Hospital	0.58	0.51	0.46	0.58
Holy Child Catholic Hospital, Fijai	1.00	0.83	0.70	0.99
Holy Family Hospital, Techiman	1.00	0.80	0.65	0.99
Holy Family Hospital, Nkawkaw	1.00	0.83	0.71	0.99
Holy Family Hospital, Berekum	1.00	0.83	0.73	0.99
Holy Spirit Clinic, Dantano	1.00	0.77	0.52	0.99
HopeXchange Medical Centre	0.40	0.36	0.33	0.40
Immaculate Conception Health Centre, Kaleo	0.56	0.48	0.41	0.55
Janie Speaks A.M.E Zion Hospital, Afrancho	0.42	0.36	0.30	0.41
Kom Presbyterian Clinic	0.35	0.30	0.25	0.35
LivingSpring Baptist Medical Centre-Atasomanso	0.07	0.06	0.06	0.07
Manna Mission Hospital	1.00	0.81	0.68	0.99
Margaret Marquart Catholic Hospital	1.00	0.82	0.67	0.99
Martyrs Of Uganda Health Centre, Bole	0.69	0.60	0.52	0.68
Mary Ekuba Ewoo SDA Clinic, Akwidaa	0.92	0.78	0.67	0.91
Mater Ecclesiae Hospital, Sokode	0.38	0.33	0.29	0.38
Mathias Catholic Hospital, Yeji	1.00	0.83	0.69	0.98
Mercy Women's Catholic Hospital	1.00	0.83	0.71	0.99
Methodist Medical Center, Adum	0.48	0.41	0.34	0.47
Bebu Methodist Clinic	0.37	0.31	0.27	0.36
Methodist Medical Centre, Apagya	0.85	0.72	0.62	0.84
Methodist Medical Centre, Brodekwano	1.00	0.77	0.55	0.99
Methodist Medical Centre, Dagyamen	1.00	0.81	0.63	0.99
Methodist medical Centre, Kwakuanya	1.00	0.79	0.63	0.99
SDA Hospital, Namong	0.66	0.58	0.53	0.66
Our Lady of Grace Hospital, Breman-Asikuma	0.92	0.80	0.71	0.90
Pentecost Clinic—Kasapin	0.53	0.44	0.38	0.52
Pentecost Hospital, Madina	1.00	0.78	0.56	0.99
Pentecost Hospital, Tarkwa	0.87	0.77	0.70	0.86
Pope Francis Health Centre, Komfourkrom	1.00	0.78	0.54	0.99
Pope John Paul II Medical Centre	0.46	0.40	0.35	0.46
Powerhouse Hospital	1.00	0.77	0.59	0.99
Presbyterian Health Centre, Assin Nsuta	1.00	0.78	0.57	0.99
Presbyterian PHC, Salaga	0.09	0.08	0.07	0.09
Presbyterian Health Centre, Kwamesua	1.00	0.82	0.68	0.99
Presbyterian Health Centre, Kyeremasu	1.00	0.77	0.55	0.99
Presbyterian Health Centre, Sumanduri	1.00	0.77	0.55	0.99
Presbyterian Clinic-Antwirifo	1.00	0.82	0.64	0.99
Presbyterian Health Centre, Papueso	0.88	0.75	0.64	0.87
Presbyterian Regional Eye Centre, Yorogo	1.00	0.77	0.53	0.99
Presbyterian PHC, Loloto	1.00	0.82	0.70	0.99
Presbyterian Health Centre, Langbinsi	1.00	0.77	0.55	0.99
Presbyterian CHPS, Amonie	1.00	0.79	0.61	0.98
Presbyterian Health Centre, Enchi	1.00	0.86	0.75	0.98
Presbyterian Health Centre, Jankufa	1.00	0.77	0.53	0.99
Presbyterian Health Centre, Kwadwokumikrom	1.00	0.77	0.53	0.99
Presbyterian Hospital, Dormaa Ahenkro	0.92	0.79	0.69	0.91
Queen of Peace Clinic, Sabuli	1.00	0.79	0.64	0.99
Richard Novati Catholic Hospital	1.00	0.77	0.53	0.99
Sacred Heart Catholic Hospital	0.99	0.86	0.76	0.98
Salvation Army Health Centre—Adaklu—Sofa	1.00	0.78	0.55	0.99
Salvation Army Health Centre, Ajumako Ochiso	1.00	0.83	0.71	0.99
Saviour Community Hospital, Bonwire	1.00	0.80	0.66	0.99
SDA Clinic and Maternity, Sefwi Punikrom	0.37	0.31	0.27	0.36
SDA clinic Denkyira Domimase	0.50	0.43	0.37	0.50
Seventh Day Adventist Clinic- Dadieso	1.00	0.81	0.66	0.99
SDA Clinic—WA	0.74	0.64	0.58	0.73
SDA Hospital Asamang	0.52	0.46	0.41	0.52
SDA Hospital, Koforidua	1.00	0.89	0.81	0.99
SDA Hospital, Dwinase	1.00	0.85	0.75	0.99
SDA Hospital, Obuasi	1.00	0.84	0.74	0.99
SDA Hospital, Sunyani	0.95	0.83	0.76	0.94
SDA Hospital, Tamale	1.00	0.78	0.61	0.98
SDA Hospital, Wiamoase	0.82	0.70	0.59	0.81
Valley View Adventist Hospital, Oyibi	0.35	0.32	0.29	0.35
SDA Hospital, Kwadaso-Kumasi	1.00	0.78	0.60	0.99
SDA Hospital, Gbawe	0.41	0.36	0.34	0.40
Grace Spring Mission Hospital	0.66	0.58	0.52	0.65
St. Dominic Clinic, Cherembo	0.67	0.57	0.44	0.66
St. Edward's Hospital, Dwinyama	0.55	0.47	0.42	0.54
St. James Clinic	0.94	0.80	0.69	0.93
St. John Health Centre, Akim Ofoase	0.38	0.32	0.27	0.38
St. Joseph's Hospital, Koforidua	0.65	0.57	0.50	0.64
St. Lucas Hospital, Wiaga	0.46	0.40	0.35	0.45
St. Mary Theresa Hospital	1.00	0.85	0.75	0.99
St. Mary's Hospital, Drobo	1.00	0.78	0.53	0.99
St. Michael's Hospital, Pramso	0.90	0.79	0.71	0.89
St Peter's Hospital—Jacobu	0.52	0.46	0.43	0.51
St. Alban's Clinic (The Refugee Camp)	1.00	0.78	0.60	0.98
St. Andrew's Catholic Hospital, Kordiabe	0.36	0.31	0.28	0.36
St. Anne's Polyclinic	0.27	0.23	0.21	0.27
St. Anthony's Clinic	0.42	0.35	0.28	0.41
St. Anthony's Hospital	1.00	0.77	0.58	0.99
St. Dominic Hospital, Akwatia	1.00	0.80	0.67	0.98
St. Elizabeth Hospital, Hwidiem	1.00	0.79	0.62	0.99
St. Francis Xavier Hospital	1.00	0.83	0.73	0.99
St. Georges Clinic, Liati	0.35	0.29	0.24	0.34
St. Gregory Catholic Hospital, Gomoa Budumburam	1.00	0.83	0.71	0.99
St. Ignatius Health Centre, Lassia Tuolu	0.77	0.65	0.57	0.76
St. John of God Hospital, Sefwi-Asafo	1.00	0.85	0.74	0.98
St. John of God Hospital, Amrahia	0.53	0.44	0.38	0.52
St. John of God Hospital, Duayaw-Nkwanta	0.99	0.85	0.73	0.97
St. Joseph’s Clinic-Wenchi Koasi	1.00	0.78	0.61	0.99
St. Joseph Health centre, Nakolo	0.57	0.50	0.46	0.56
St. Joseph's Hospital, Nkwanta, Oti	0.49	0.44	0.41	0.48
St. Joseph's Clinic, Bechem	0.03	0.03	0.02	0.03
St. Luke's Clinic, Chinderi	0.45	0.39	0.34	0.45
St. Luke catholic hospital, apam	0.95	0.83	0.74	0.94
St. Marks Anglican Clinic	1.00	0.78	0.53	0.99
St. Martin de Porres Hospital, Eikwe	1.00	0.80	0.67	0.98
St. Martin Memorial Hospital—Dansoman	0.55	0.49	0.44	0.55
St. Martin's Catholic Hospital, Agroyesum	0.73	0.63	0.56	0.72
St. Martins Memorial Hospital—Shukura	1.00	0.78	0.53	0.98
St. Martin's Memorial Hospital—Ashaiman	0.76	0.69	0.65	0.75
St. Matthew's hospital, ampenkro	1.00	0.84	0.73	0.99
St. Patrick's Hospital, Offinso	1.00	0.81	0.71	0.99
St. Theresa's hospital, Nkoranza	0.87	0.75	0.65	0.86
St. Theresa's Hospital, Nandom	1.00	0.78	0.60	0.99
St. Vincent DePaul Clinic, Drobonso	0.56	0.48	0.40	0.55
St. Christopher Health Centre, Dapouri	1.00	0.77	0.54	0.99
St. Joseph's Hospital, Jirapa	0.77	0.67	0.59	0.77
St. Martin's De Porres Health Centre, Eremon	0.80	0.68	0.60	0.79
St. Stella's Clinic, Karni	1.00	0.77	0.55	0.99
Presbyterian Health Centre, Suma Ahenkro	1.00	0.77	0.55	0.99
Tanoah Baptist Medical Centre, Opuniase	0.43	0.37	0.28	0.43
Tatale district hospital, tatale	0.89	0.78	0.71	0.88
Salvation Army Hospital, Wiamoase	1.00	0.80	0.67	0.99
Todah Hospital, Obuasi	1.00	0.78	0.55	0.99
True Faith Hospital—Kumawu Bodomase	0.25	0.22	0.17	0.25
Valley View Adventist Hospital, Techiman	0.72	0.63	0.55	0.71
St. Anne's Hospital, Damongo	0.55	0.48	0.44	0.54
Presbyterian Health Centre, Widana	1.00	0.80	0.66	0.99

## Abbreviations

GDP: Gross Domestic Product
UHC: Universal Health Coverage
LMICs: Lower Middle Income Countries
CHAG: Christian Health Association of Ghana
DMUs: Decision Making Units
DEA: Data Envelopement Analysis
CRS: Constant Returns to Scale
VRS: Variable Returns to Scale
PTE: Pure Technical Efficiency
SE: Scale Efficiency
DRS: Decreasing Returns to Scale
IRS: Increasing returns to scale
TE: Technical Efficiency
SE: Scale Efficiency
PTE: Pure Technical Efficiency
PCH: Primary Care Hospitals
SCH: Secondary Care Hospitals
OLHF: Other Lower Level Health Facilities
CHPS: Compounds and Health Centres
ANC: Antenatal Care

## References

[1] Mbau R, Musiega A, Nyawira L, Tsofa B, Mulwa A, Molyneux S, et al. Analysing the efficiency of health systems: a systematic review of the literature. Appl Health Econ Health Policy. 2023;21(2):205–24. 36575334 10.1007/s40258-022-00785-2 PMC9931792

[2] Jakovljevic M, Timofeyev Y, Ekkert NV, Fedorova JV, Skvirskaya G, Bolevich S, et al. The impact of health expenditures on public health in BRICS nations. J Sport Heal Sci. 2019;8(6):516. 10.1016/j.jshs.2019.09.002 PMC6835015 31720060

[3] Corporation IF. The Business of Health in Africa: Partnering with the private sector to improve people’s lives. Washingt (District Columbia) Int Financ Corp. 2007.

[4] Jakovljevic M, Getzen TE. Growth of global health spending share in low and middle income countries. Front Pharmacol. 2016;7:21. 26903867 10.3389/fphar.2016.00021 PMC4751681

[5] Babalola TK, Moodley I. Assessing the efficiency of health-care facilities in Sub-Saharan Africa: a systematic review. Heal Serv Res Manag Epidemiol. 2020;7:2333392820919604. 10.1177/2333392820919604 PMC7218466 32426420

[6] Nassar H, Sakr H, Ezzat A, Fikry P. Technical efficiency of health-care systems in selected middle-income countries: an empirical investigation. Rev Econ Polit Sci. 2020;5(4):267–87. 10.1108/REPS-03-2020-0038

[7] World Health Organization. Technical efficiency of health systems in the WHO African Region. 2021.

[8] Cylus J, Papanicolas I, Smith PC. A framework for thinking about health system efficiency. Heal Syst Effic. 2016;1:3.

[9] Zere E, Mbeeli T, Shangula K, Mandlhate C, Mutirua K, Tjivambi B, et al. Technical efficiency of district hospitals: evidence from Namibia using data envelopment analysis. Cost Eff Resour Alloc. 2006;4(1):1–9. 16566818 10.1186/1478-7547-4-5 PMC1524815

[10] Novignon J, Nonvignon J. Improving primary health care facility performance in Ghana: efficiency analysis and fiscal space implications. BMC Health Serv Res. 2017;17:1–8. 28606131 10.1186/s12913-017-2347-4 PMC5468971

[11] Akazili J, Adjuik M, Jehu-Appiah C, Zere E. Using data envelopment analysis to measure the extent of technical efficiency of public health centres in Ghana. BMC Int Health Hum Rights. 2008;8(1):11. 19021906 10.1186/1472-698X-8-11 PMC2605432

[12] Jehu-Appiah C, Sekidde S, Adjuik M, Akazili J, Almeida SD, Nyonator F, et al. Ownership and technical efficiency of hospitals: evidence from Ghana using data envelopment analysis. Cost Eff Resour Alloc. 2014;12(1):9. 24708886 10.1186/1478-7547-12-9 PMC4108084

[13] Zere E, Kirigia JM, Duale S, Akazili J. Inequities in maternal and child health outcomes and interventions in Ghana. BMC Public Health. 2012. 10.1186/1471-2458-12-252 PMC3338377 22463465

[14] Akazili J, Adjuik M, Chatio S, Kanyomse E, Hodgson A, Aikins M, et al. What are the technical and allocative efficiencies of public health centres in Ghana? Ghana Med J. 2008;42(4):149. 19452023 PMC2673839

[15] Osei D, d’Almeida S, George MO, Kirigia JM, Mensah AO, Kainyu LH. Technical efficiency of public district hospitals and health centres in Ghana: a pilot study. Cost Eff Resour Alloc. 2005;3(1):1–13. 16188021 10.1186/1478-7547-3-9 PMC1253524

[16] Christian Health Association of Ghanna. Annual Report 2021. Accra, Ghana; 2022.

[17] Abekah-Nkrumah G, Ofori CG, Antwi M, Attachey AY, Wit TFR de, Janssens W, et al. Effect of the Implementation of Med4All Platform on Business Performance of Selected Health Facilities in Ghana: Baseline Report. Accra; 2023.

[18] Verbeke F, Ndabaniwe E, Van Bastelaere S, Ly O, Nyssen M. Evaluating the impact of hospital information systems on the technical efficiency of 8 Central African hospitals using Data Envelopment Analysis. J Heal Inform Afr. 2013;1(1):11–20.

[19] Verbeke F, Karara G, Nyssen M. Evaluating the impact of ICT-tools on health care delivery in sub-Saharan hospitals. Stud Health Technol Inform. 2013;192:520–3. 23920609

[20] Lee J, McCullough JS, Town RJ. The impact of health information technology on hospital productivity. RAND J Econ. 2013;44(3):545–68. 10.1111/1756-2171.12030

[21] Kohl S, Schoenfelder J, Fügener A, Brunner JO. The use of Data Envelopment Analysis (DEA) in healthcare with a focus on hospitals. Health Care Manag Sci. 2019;22(2):245–86. 29478088 10.1007/s10729-018-9436-8

[22] Farrell MJ. The measurement of productive efficiency. J R Stat Soc Ser A. 1957;120(3):253–81. 10.2307/2343100

[23] Charnes A, Cooper WW, Rhodes E. Measuring the efficiency of decision making units. Eur J Oper Res. 1978;2(6):429–44. 10.1016/0377-2217(78)90138-8

[24] Banker RD, Charnes A, Cooper WW. Some models for estimating technical and scale inefficiencies in data envelopment analysis. Manage Sci. 1984;30(9):1078–92. 10.1287/mnsc.30.9.1078

[25] Turkson C, Liu W, Acquaye A. A data envelopment analysis based evaluation of sustainable energy generation portfolio scenarios. Appl Energy. 2024;363: 123017. 10.1016/j.apenergy.2024.123017

[26] Ramanathan R. An introduction to data envelopment analysis: a tool for performance measurement. Sage; 2003.

[27] Abekah-Nkrumah G, Abor PA. Engagement of the private health sector in the delivery of Ghana’s COVID-19 Emergency Response. Congo Brazaville; 2022. 10.1136/bmjgh-2023-014217 PMC11912757 39694620

[28] Abekah-Nkrumah G, Dumenu MY, Asante K, Armah-Attoh D, Dome MZ, Essima LO, et al. Impact Of Covid-19 on Government’s Reform Programmes in Ghana [Internet]. Accra; 2021. (Prepared by Ghana Center For Democratic Development (CDD-Ghana) and Published by Deutsche Gesellschaft für Internationale Zusammenarbeit (GIZ) GmbH). Available from: https://cddgh.org/wp-content/uploads/2021/12/CDD-GIZ_COVID-19-Studies-Ghana.pdf

[29] Abekah-Nkrumah G, Antwi M, Attachey AY, Janssens W, Rinke de Wit TF. Readiness of Ghanaian health facilities to deploy a health insurance claims management software (CLAIM-it). PLoS ONE. 2022;17(10): e0275493. 36197932 10.1371/journal.pone.0275493 PMC9534449

[30] CITI News Room. CHAG to make savings from drug procurement through CHAG–PharmAccess Med4All digital platform. 2022. Available from: https://citinewsroom.com/2022/03/chag-to-make-savings-from-drug-procurement-through-chag-pharmaccess-med4all-digital-platform/

[31] Myjoyonline. NHIA commends CHAG, PharmAccess, and HeFRA for promoting quality healthcare in Ghana. 2023. Available from: http://myjoyonline.com/nhia-commends-chag-pharmaccess-and-hefra-for-promoting-quality-healthcare-in-ghana/

[32] National health Insurance Authority (NHIA). NHIA clarifies issues raised by the Ranking Member on the Parliamentary Select Committee on Health | 5/11/2022. 2023. Available from: https://www.nhis.gov.gh/News/nhia-clarifies-issues-raised-by-the-ranking-member-on-the-parliamentary-select-committee-on-health-5391

[33] Zihindulai G, MacGregor RG, Ross AJ. A rural scholarship model addressing the shortage of healthcare workers in rural areas. South Afr Heal Rev. 2018;2018(1):51–7.

[34] Anyangwe SCE, Mtonga C. Inequities in the global health workforce: the greatest impediment to health in sub-Saharan Africa. Int J Environ Res Public Health. 2007;4(2):93–100. 17617671 10.3390/ijerph2007040002 PMC3728573

[35] Okyere E, Mwanri L, Ward P. Is task-shifting a solution to the health workers’ shortage in Northern Ghana? PLoS One. 2017;12(3): e0174631. 28358841 10.1371/journal.pone.0174631 PMC5373592

